# Shear-Lag Attenuation of Interfacial Damage Kinetics in Aligned Carbon Nanotube/Polydimethylsiloxane Composites

**DOI:** 10.3390/ma19194124

**Published:** 2026-09-26

**Authors:** Hassan Syed Tamzid, Jung-Hoon Yun

**Affiliations:** 1 Department of Future Convergence Engineering, Kongju National University, Cheonan 31080, Republic of Korea; syedtamzid000@gmail.com; 2 Global Institute of Manufacturing Technology (GITECH), Kongju National University, Cheonan 31080, Republic of Korea

**Keywords:** carbon nanotube/polydimethylsiloxane composite, shear-lag homogenization, Krenchel orientation factor, interfacial damage, cyclic stiffness degradation, closed-form attenuation map

## Abstract

Aligned carbon nanotube/polydimethylsiloxane (CNT/PDMS) composites soften under repeated loading, but the microscopic rate of interfacial degradation has not been related in closed form to the macroscopic rate of stiffness loss. This paper derives that relation for a Krenchel-type, orientation-averaged Cox shear-lag homogenization whose interfacial load-transfer efficiency decays exponentially with cycle number at a prescribed rate *κ*. Because that efficiency enters the modulus nonlinearly—under a square root, then a saturating length-efficiency function—the instantaneous macroscopic decay rate is *κ* times an exact dimensionless sensitivity function *S*(*x*) of one operating point, *x* = *βs*, the shear-lag parameter times the aspect ratio. S is proved to be a strictly decreasing bijection from (0, ∞) onto (0, 1) with closed-form limits, so the mapping inverts uniquely; the rate apparent over a finite window is likewise exact, so the attenuation depends on the window length as well as the operating point. At the illustrative operating points, a least-squares rate fitted to sparse checkpoints understates the interfacial rate by a factor of about three, and the exact 50-cycle window-averaged rate understates it by a factor of up to 6.32. Both scale with the prescribed, uncalibrated shear-lag prefactor, and the fitted-constant factor exceeds 1.4 throughout. All model-derived results are analytical; no experimental validation is claimed.

## 1. Introduction

Load transfer in a discontinuously reinforced composite is controlled by shear at the inclusion–matrix interface. The Cox shear-lag analysis [1] and the Krenchel orientation factor [2] together provide the standard closed-form description of that transfer, relating the effective axial modulus to the inclusion aspect ratio, the orientation distribution, and the volume fraction; Nairn [3] has set out the assumptions under which the shear-lag kernel remains quantitatively defensible. The same framework has been carried over to nanotube-reinforced polymers, where it competes with Eshelby–Mori–Tanaka and Halpin–Tsai homogenization [4,5]. In every case, the interface is treated as a fixed property of the material. That assumption is reasonable for monotonic loading and untenable for a component in service, where the interface is the element most exposed to progressive damage.

This paper addresses the consequence of relaxing that assumption. If the interfacial coupling efficiency decays with cycle number, at what rate does the macroscopic modulus follow? The question matters practically because the quantity an experimentalist measures—a decay constant fitted to the secant modulus at a handful of cycle checkpoints—is not the quantity that governs the interface. The two are shown below to be related by an exact attenuation function of a single dimensionless operating point, and at the operating points examined here, the discrepancy between them is a factor of about three over a sparse checkpoint schedule and larger still over shorter measurement windows.

The material system used to evaluate the map is aligned multi-walled carbon nanotube (MWCNT) reinforcement in a polydimethylsiloxane (PDMS) matrix. PDMS is a compliant elastomer whose cured, unfilled Young’s modulus is only about 1 MPa [6], so the reinforcement contrast with a nanotube bundle is extreme, and the shear-lag length efficiency sits far from its saturated limit—precisely the regime in which the attenuation derived below is strongest. Composites of this class are of independent engineering interest across a range of load-bearing and compliant-structure contexts [7,8,9,10]. Aligned-nanotube (A-CNT) architectures are attractive mechanically because alignment raises the stiffness anisotropy of the composite [11]. The electrical response of carbon nanotube networks is separately sensitive to ambient conditions: the room-temperature gas-sensing response of CNT devices is measurably affected by ambient humidity [12]. That sensitivity is one reason the treatment developed here is restricted to elastic stiffness (Section 4.3). In service, such components accumulate thousands to millions of load cycles, so the stability of the as-fabricated stiffness is a design requirement rather than a curiosity.

Three coupled morphological parameters govern reinforcement of a soft matrix by nanotubes: filler volume fraction *V*_f_, inclusion aspect ratio *L*/*d*, and the orientation distribution of the inclusions relative to the loading axis [13]. Substantial micromechanical work has predicted the longitudinal and transverse moduli of A-CNT/PDMS using Eshelby–Mori–Tanaka homogenization [14,15], Halpin–Tsai laminate theory [16], and Cox–Krenchel shear-lag models [1,2]. This body of work is almost entirely confined to the linear-elastic response under monotonic loading; the systematic evolution of stiffness under repeated cycling has received little attention.

Cyclic softening of filled elastomers is itself well documented. The Mullins effect—an irreversible softening over the first few cycles before a plateau is reached—has been attributed to chain disentanglement, irreversible interfacial damage, disruption of filler agglomerates, and, in some elastomers, covalent bond scission [17,18,19,20]. Cantournet et al. [17] modeled this softening as frictional dissipation between elastomeric chains and filler surfaces, and coarse-grained molecular dynamics has reproduced its principal features [21]. PDMS/MWCNT films exhibit stiffness changes of up to 35% during the first few dozen cycles, the direction depending on strain amplitude and rate [22,23]; at higher cycle counts, an anomalous stiffening following the initial softening has also been reported [24]. Comparable behavior is documented for carbon black, silica, graphene, and clay compounds [25,26,27], suggesting that similar physics governs all filled elastomers in which filler and matrix are not covalently bonded.

What is missing from this literature is the link between the two levels. The Mullins-effect models describe damage at the interface; the micromechanical models describe reinforcement by an intact interface. Joining them analytically shows that the macroscopic observable is a strongly attenuated and cycle-dependent image of the microscopic process and that the attenuation factor is fixed by a single dimensionless shear-lag operating point, into which the inclusion geometry and the prescribed constituent parameters enter together.

The contribution of this paper is analytical. Section 2 assembles the Cox–Krenchel homogenization, the Krenchel orientation average, and a prescribed exponential interfacial-damage law into a single closed-form expression for the cycle-dependent axial modulus; Section 3 develops its consequences. Four results follow directly from those equations and are independent of any numerical dataset. (i) The instantaneous macroscopic decay rate is the prescribed microscopic rate multiplied by an exact dimensionless sensitivity function *S*(*x*) of one dimensionless operating point, *x* = *βs*. (ii) *S* is a strictly decreasing bijection from (0, ∞) onto (0, 1), with closed-form small- and large-argument limits, so the mapping between the microscopic and macroscopic rates admits a unique inverse. (iii) The apparent rate obtained over a finite measurement window has an exact closed form and is a cycle-averaged sensitivity, so the attenuation depends on the length of the measurement window as well as on geometry. (iv) At bundle aspect ratios representative of aligned nanotube forests, the attenuation is a factor of about three, so an interfacial damage constant read directly from a macroscopic stiffness decay is understated by that factor unless the correction is applied.

**Scope of this study.** This paper is a theoretical contribution. The interfacial-damage constant *κ* and the shear-lag prefactor *k*_l_ are prescribed model inputs, not quantities calibrated against measurement, and the exponential form of the damage law is imposed rather than derived. No new experimental data are reported, and no experimental validation is claimed. Numerical values in this paper are of exactly three kinds, and the distinction is maintained throughout: quantities **derived** analytically from Equations (1)–(9); parameters **prescribed** as model inputs (Table 1); and **illustrative** calculations obtained by evaluating those equations at a stated nominal operating point, identified as illustrative wherever they appear. Section 5 states what is and is not established by the framework, and Section 4.4 sets out the measurements that would be required to calibrate it.

## 2. Analytical Framework

### 2.1. Composite Representation and Constituent Parameters

The composite is represented as a population of straight, rigid, cylindrical inclusions embedded in an isotropic linear-elastic matrix and oriented about a single axis. Each inclusion represents a densified MWCNT bundle rather than a single tube, reflecting the clustering that occurs when neighboring 10–20 nm tubes coalesce during chemical vapor deposition (CVD) on patterned catalyst substrates [6,28]. The bundle length is *L* = 15 µm throughout.

All illustrative evaluations in this paper are carried out at a single nominal operating point, defined by two filler populations that differ in bundle count and bundle coarseness (Table 1; Figure 1a). The dilute population (*n*_CNT_ = 250 bundles in a cubic domain of edge 20 µm, *V*_cell_ = 8 × 10^−15^ m^3^) consists of finer bundles of effective diameter *d*_eff_ = 100 nm; the concentrated population (*n*_CNT_ = 400) consists of coarser bundles of *d*_eff_ = 130 nm. Every quantity named in the preceding sentences is a prescribed model input, listed with its value, units, and status in Table 1. These are the nominal inputs of the authors’ own earlier, unpublished modeling of the same system, restated here unchanged and in full; none is inferred from measurement, from a fit, or from the output of any previously generated dataset. The bundle volume and the volume fraction follow from that prescribed geometry alone, *V*_bundle_ = π*d*_eff_^2^*L*/4 and *V*_f_ = *n*_CNT_*V*_bundle_/*V*_cell_, giving *V*_bundle_ = 1.178 × 10^−19^ and 1.991 × 10^−19^ m^3^ and *V*_f_ = 0.368% and 0.995%, respectively. Because bundle count and bundle diameter co-vary, *n*_CNT_ is a composite loading descriptor rather than an independently tunable variable. The mechanically decisive consequence is that the aspect ratio *s* = *L*/*d*_eff_ differs between the two populations, taking the values 150.0 and 115.4; it is this aspect ratio that carries most of the difference between their dimensionless operating points (Section 2.4 and Table 2).

The PDMS matrix is treated as a homogeneous, isotropic, linear-elastic solid of undeformed Young’s modulus *E*_m_ = 1.0 MPa, consistent with characterization data for cured Sylgard-184 (Dow, Midland, MI, USA) [6,23]. The constituent mass densities, 1900 kg m^−3^ for the nanotube phase [6] and 970 kg m^−3^ for the matrix, are recorded for completeness of the material description; neither enters Equations (1)–(9), which are formulated entirely in terms of volume fraction. The inclusion phase enters through an effective bundle modulus *E*_CNT_ = 0.4 GPa, far below the intrinsic single-tube value of ≈1 TPa. The reduction reflects inter-tube slip within the densified bundles and the bundle waviness that assumption A1 does not resolve; both are absorbed into this prescribed effective value. Bundle-scale moduli of this order are consistent with the stochastic morphological analysis of aligned nanotube arrays by Stein et al. [28]. Like *κ* and *k*_l_, *E*_CNT_ is a prescribed effective bundle-scale model parameter rather than a calibrated one, and its influence is bounded: it enters only Equation (2) and the classical bound on *k*_l_ in Section 2.3, so it scales the absolute moduli evaluated in Section 3.6 but appears nowhere in the attenuation results of Section 3.1, Section 3.2, Section 3.3, Section 3.4 and Section 3.5. Both volume fractions lie in the dilute regime relevant to elastomeric nanocomposites; percolation of an electrically conducting nanotube network [29] lies outside the scope of this elastic-mechanical treatment.

The formulation solves no boundary-value problem on the domain: the effective modulus follows from a closed-form orientation average (Section 2.3), so the domain functions only as the statistical volume element over which orientations and volumes are defined. It is not a representative volume element in the field-resolved sense, and no traction or displacement condition is imposed on its faces.

### 2.2. Orientation Averaging

The director of each bundle makes an angle *θ* with the axial loading direction *z*, distributed as a zero-mean Gaussian in the polar angle with standard deviation *σ*_θ_ (Figure 1b). The distribution is applied to *θ* alone; no azimuthal component or sin *θ* solid-angle weighting is included, so the orientation state is planar rather than a full three-dimensional orientation distribution function [13]. The Gaussian is taken on the whole line; because cos^4^*θ* has period π, this is equivalent to wrapping it onto (−π/2, π/2], and Equation (1) is exact for either statement. The Krenchel orientation factor is the fourth moment of the director cosine, for which the Gaussian closure gives a closed form:

*η*_o_ = ⟨cos^4^*θ*⟩ = 3/8 + ½ exp(−2*σ*_θ_^2^) + ⅛ exp(−8*σ*_θ_^2^) (1)

where *σ*_θ_ is expressed in radians. Over the window *σ*_θ_ = 25–45° used for illustration, Equation (1) gives *η*_o_ = 0.744, 0.678, 0.618, 0.566, and 0.522, respectively. The window spans a moderately well-aligned CVD forest at its lower bound and a substantially misaligned but still anisotropic state at its upper bound. *σ*_θ_ = 45° does not correspond to a disordered material: a planar-random distribution gives ⟨cos^4^*θ*⟩ = 3/8 = 0.375, and a three-dimensional random distribution gives 1/5 = 0.200, both well below 0.522. Every condition considered here is therefore an aligned architecture, and no result should be extrapolated to dispersion-cast or otherwise randomly oriented composites.

### 2.3. Shear-Lag Homogenization with a Cycle-Dependent Interface

The effective axial modulus is obtained from a Krenchel-type, orientation-averaged shear-lag (modified rule-of-mixtures) homogenization, in which the bundle population reinforces the matrix in proportion to the orientation factor, the cycle-dependent Cox length efficiency, and the bundle volume fraction:

*E*(*N*) = (1 − *V*_f_) *E*_m_ + *η*_o_ *η*_l_(*N*) *V*_f_ *E*_CNT_ (2)

where *E*_m_ and *E*_CNT_ are the Young’s moduli of the matrix and of the inclusion phase. Because *V*_f_ ≪ 1, the reinforcing term diminishes as the interface degrades and the modulus relaxes toward the asymptote *E*_∞_ = (1 − *V*_f_)*E*_m_, recovering the constrained matrix stiffness in the fully decoupled limit.

The cyclic interfacial state is condensed into a single scalar coupling efficiency *η*_int_(*N*) ∈ (0, 1], prescribed to decay exponentially with cycle number:

*η*_int_(*N*) = exp(−*κN*) (3)

where *κ* = 3.087 × 10^−2^ cycle^−1^ is a prescribed, uniform microscopic interfacial-damage rate constant that sets the cyclic timescale of the model. Like *k*_l_ below, it is an inherited nominal value, taken from the authors’ own earlier, unpublished modeling of this system and carried over unchanged, and the figures it carries are not significant digits of a measurement. It is not calibrated against measurement, and Equation (3) is imposed rather than derived; both points are carried forward in Section 4 and Section 5, and what inherits the value of *κ* is stated in Section 5.3. The coupling efficiency enters the modulus through the shear-lag parameter and the Cox length efficiency:

*β*(*N*) = *k*_l_ [*η*_int_(*N*)/(1 − *V*_f_)]^1/2^ = *β*_0_ exp(−*κN*/2),     *x*(*N*) = *β*(*N*)*s*,     *η*_l_(*N*) = 1 − tanh *x*(*N*)/*x*(*N*) (4)

where *s* = *L*/*d*_eff_ is the bundle aspect ratio and *k*_l_ is a dimensionless shear-lag prefactor. Both *k*_l_ and *β* are dimensionless: *β* is the classical shear-lag parameter nondimensionalized by the effective bundle radius, so that the argument *x* = *βs* reproduces the classical Cox grouping formed from a dimensional shear-lag parameter and the inclusion half-length. The length efficiency therefore decreases with cycling rather than remaining constant, and the reinforcement enters the modulus through this nonlinear, saturating term. It is this nesting—a square root followed by a saturating tanh—that produces the attenuation derived in Section 2.4; the full chain from *κ* to *E*(*N*) is summarized in Figure 1c.

***Status of the prefactor k*_l_**. The value used for illustration, *k*_l_ = 0.042, is a prescribed phenomenological model parameter; it is intended to represent the stress-transfer efficiency across a compliant PDMS interphase in a lumped way. How it was chosen can be stated exactly: it was not chosen by any procedure reported here, and it was not chosen to fit, match, or reproduce anything. It is an inherited nominal modeling choice—the operating value the authors had adopted in their own earlier, unpublished modeling of this system—retained here unchanged so that the illustrative evaluations remain comparable with it. That earlier modeling is not cited, and no measured or calibrated origin for the value is claimed; its retention is a matter of continuity in the operating point, not of evidence. No procedure in this paper calibrates, fits, or optimizes *k*_l_; it is a nominal, uncalibrated model input. It is not an experimentally identified material constant of CNT/PDMS; it is not a property of any specimen, and it should not be read as a Cox shear-lag constant transferable to another system. For comparison, the classical Cox prefactor for dilute elastic fibers, *k*_l_ = [2*G*_m_/(*E*_CNT_ ln(*R*/*r*))]^1/2^ with *G*_m_ = *E*_m_/2(1 + *ν*) = 0.33 MPa and *E*_CNT_ = 0.4 GPa, ranges from 0.017 to 0.028 at the present volume fractions (0.0172 to 0.0276 before rounding). The span is generated by the four conventions in standard use for the fiber-spacing logarithm ln(*R*/*r*) [1,3]—hexagonal packing, ln(*R*/*r*) = ½ln [2π/(√3*V*_f_)]; square packing in its two forms, ½ln(π/*V*_f_) and ½ln[π/(4*V*_f_)]; and the dilute logarithmic form ln(*R*/*r*) = ln(1/*V*_f_)—each evaluated at both volume fractions. The reference implementation in the Supplementary Materials (Section S2) prints all eight values. The interval 0.019–0.027 used in Table 3 and in the shaded bands of Figure 2a and of the design map in Section 3.5 is a rounded interior interval of that span, retained unchanged from the earlier version of this manuscript; it is not the pair of endpoints of any single convention. The value used here therefore lies above the classical range under every convention. It is reported as a prescribed constant of exactly the same epistemic status as *κ*, and the consequences of the choice are quantified rather than assumed away: because the attenuation map of Section 2.4 depends on *k*_l_ only through the operating point *x*_0_ = *β*_0_*s*, the full sensitivity to *k*_l_ across and above the classical range is tabulated in Section 3.3 (Table 3). Changing it relocates the evaluation on the fixed curve *S*(*x*)—altering the entries of Table 2 and Table 4 and the aspect-ratio thresholds of Section 3.5—but leaves Equation (6) and every property proved for it in Section 3.1 untouched, so the attenuation mechanism derived here does not depend on the numerical choice at all. The qualitative conclusion of this paper survives across that whole range; the numerical factor does not and is not presented as a material property.

The loading idealization is a spatially uniform uniaxial extension along the alignment axis at 1% strain amplitude under a rate-independent, quasi-static assumption. Because the constitutive response of both phases is linear elastic, the secant modulus is independent of the strain interval over which it is taken, and no stress–strain history is integrated. The small-strain, rate-independent setting is a deliberate restriction of scope, not an approximation to large-strain elastomer behavior; its consequences are stated in Section 4.2 and Section 5.3.

### 2.4. The Attenuation Map

Because *η*_int_ enters the modulus through *β* under a square root and then through the saturating tanh of *η*_l_, the macroscopic modulus does not decay at the microscopic rate *κ*. The relation between the two can be obtained exactly. With *E*_∞_ = (1 − *V*_f_)*E*_m_, the normalized modulus is

*Ê*(*N*) = [*E*(*N*) − *E*_∞_]/[*E*_0_ − *E*_∞_] = *η*_l_[*x*(*N*)]/*η*_l_(*x*_0_) (5)

Thus, the whole cyclic response is carried by the length efficiency alone. The orientation factor cancels identically since it enters Equation (2) only as a prefactor of the reinforcing term. The volume fraction does not cancel in that sense: its explicit prefactor cancels, but *V*_f_ re-enters through *β*_0_ = *k*_l_(1 − *V*_f_)^−1/2^ and hence through the operating point *x*_0_ = *β*_0_*s*, so it survives in Equation (5) through *x*_0_ even though it does not appear there explicitly. Differentiating Equation (4) gives d*β*/d*N* = −(*κ*/2)*β*(*N*) exactly, so the shear-lag parameter always decays at precisely half the prescribed microscopic rate, independently of *β*_0_ and *s*. Writing *x*(*N*) = *β*(*N*)*s* and applying the chain rule to *η*_l_(*x*) = 1 − tanh(*x*)/*x* gives the instantaneous local logarithmic decay rate of the macroscopic modulus:

*k*_loc_(*N*) ≡ −d ln *η*_l_/d*N* = *κ* · *S*[*x*(*N*)], *S*(*x*) = [tanh *x* − *x* sech^2^*x*]/(2*x*[1 − tanh *x*/*x*]) (6)

An equivalent and more compact statement of the same result is

*S*(*x*) = (*x*/2) · d ln *η*_l_/d*x* (7)

which identifies *S* as one half of the logarithmic sensitivity of the length efficiency to the operating point. Equation (6) was verified numerically against the definition −d ln *η*_l_/d(*κN*) at *x* = 0.5, 2, 4.870, 6.312, 20, and 60. The agreement is better than 10^−49^. Because a central difference cannot resolve a discrepancy of that size in 50-digit arithmetic, this particular check is carried out at 140 digits, as shown by the reference implementation in the Supplementary Materials (Section S2), which prints the bound attained.

Three properties of *S* follow directly and are established in Section 3.1: it is bounded in (0, 1); it is strictly decreasing on (0, ∞), hence a bijection onto (0, 1) whose inverse is single-valued; and both of its limits are available in closed form. Consequently, *k*_loc_(*N*) ≤ *κ* at every cycle count, with equality only in the fully decoupled limit, and a measured local rate determines the operating point uniquely through *x* = *S*^−1^(*k*_loc_/*κ*). Within assumptions A1–A8, the functional form of the attenuation depends on the single dimensionless operating point *x* = *βs* alone: Equation (6) contains no reference to *η*_o_, to *V*_f_, to *E*_CNT_, to *k*_l_, or to the absolute value of *κ*, so the curve *S*(*x*) is the same for every parameter set those assumptions admit. Those parameters enter only through the value of *x*—*k*_l_ and *V*_f_ through *β*_0_, and the inclusion geometry through *s*—and therefore select a point on a fixed curve rather than changing the curve. The distinction matters for the inversion of Section 3.1: the map is common to all of them, but the operating point at which it is evaluated is not.

**What cancels in a ratio and what does not.** Equation (6) is linear in *κ*, so for two populations of the same interface chemistry the ratio of their initial local rates, *k*_loc,1_(0)/*k*_loc,2_(0) = *S*(*x*_0,1_)/*S*(*x*_0,2_), is exactly free of *κ*. That cancellation belongs to the instantaneous rate alone and does not survive over a finite measurement window. The apparent rate of Equation (8) below depends on *κ* twice, once through the explicit factor and once through the window endpoint *x*(*N*_max_) = *x*_0_ exp(−*κN*_max_/2), and the second dependence does not cancel when two such rates are divided. At the two illustrative operating points of Table 1, the ratio *k*_app,1_/*k*_app,2_ over a 100-cycle window is 0.7593, against 0.7296 for the initial local rates, and it becomes 0.7178 or 0.8850 if *κ* is halved or doubled; it approaches the *κ*-free value 0.7296 only as the window length tends to zero. What is *κ*-free at a finite window is not the ratio but the locus: the two instances of Equation (8) share the single factor exp(−*κN*_max_/2), so eliminating it leaves one relation between *k*_app,1_ and *k*_app,2_ that contains no *κ*. That relation, and not a predicted ratio, is the falsifiable content used in Section 4.4.

### 2.5. Apparent Decay Rate over a Finite Measurement Window

Equation (6) gives an instantaneous rate, whereas an experiment returns a single constant fitted over a bounded window of cycles. The two are related exactly. Integrating Equation (6) between 0 and *N*_max_ and dividing by the window length gives the apparent (window-averaged) decay rate

*k*_app_(*N*_max_) = (1/*N*_max_) ln[*η*_l_(*x*_0_)/*η*_l_(*x*(*N*_max_))] = *κ* ⟨*S*⟩_[0,Nmax]_ (8)

in which ⟨*S*⟩ denotes the mean of *S*[*x*(*N*)] over the window. Equation (8) is exact, requires no fitting, and shows that the apparent attenuation *κ*/*k*_app_ is a property of two quantities and not one: the geometric operating point *x*_0_ and the length of the measurement window. It is the analytical counterpart of the quantity an experimentalist reports. Because *Ê*(*N*) = *η*_l_[*x*(*N*)]/*η*_l_(*x*_0_), Equation (8) may equivalently be written as *k*_app_(*N*_max_) = −ln *Ê*(*N*_max_)/*N*_max_, so the exact apparent rate is fixed by the normalized modulus at the two ends of the window and involves no regression of any kind.

Where a least-squares constant is wanted instead—because that is what is normally fitted—the single-parameter exponential $\overset{\text{-}}{k}$ is obtained by least-squares fitting of Equation (5) to a stated set of checkpoints. A four-point schedule *N* = 0, 100, 300, and 500 cycles is used for illustration throughout, chosen to mirror laboratory practice in which the secant modulus is recorded at a small number of bounded checkpoints rather than continuously. The resulting $\overset{\text{-}}{k}$ is a fit-weighted average of the monotonically increasing local rate over that schedule. It is neither a constant intrinsic to the model nor equal to the initial sensitivity *S*(*x*_0_); the two are distinct quantities and are not expected to coincide. A general closed-form expression for $\overset{\text{-}}{k}$ as a function of *κ*, *x*_0_, and an arbitrary schedule is not derived here and remains to be derived. The two summaries must not be interchanged. $\overset{\text{-}}{k}$ minimizes a sum of squared residuals over a chosen schedule and satisfies no identity of the form of Equation (8); substituting it into Equation (8) and solving for *κ* is therefore not a valid inversion. The discrepancy is not negligible: at the operating points of Table 1, the four-point $\overset{\text{-}}{k}$ happens to equal the exact *k*_app_ only at window lengths of *N*_max_ ≈ 107 and 103 cycles, and those crossing points are fixed by the chosen schedule rather than by any measurement window. Wherever *κ* is to be recovered from measurement, the exact *k*_app_ of Equation (8) is the quantity to use; $\overset{\text{-}}{k}$ is reported in this paper only as a descriptive summary of the checkpoint series.

### 2.6. Damage-Index Form

For comparison with damage-mechanics formulations, it is convenient to recast Equation (5) as a damage index *D*(*N*) = 1 − *Ê*(*N*). In the expression below, the first equality is a definition and is exact; the second holds only to the accuracy of the single-exponential summary of Section 2.5, and is written as an approximation for that reason:

(9) $$D(N)=1-\hat{E}(N)\approx 1-\exp(-\overset{\text{-}}{k}N)$$

The stiffness-reduction variable *D* ∈ [0, 1) is the classical continuum-damage measure of Kachanov [30]; the exponential right-hand side is not. That form follows from the least-squares summary adopted in Section 2.5, and [30] is cited here for the damage variable and its normalization, not for any kinetic law of damage accumulation. The exact trajectory *D*(*N*) = 1 − *η*_l_[*x*(*N*)]/*η*_l_(*x*_0_) is not a single exponential in *N* at any operating point, as Section 3.2 shows. With that scope understood, Equation (9) inherits the attenuation: the damage index evolves at the apparent rate $\overset{\text{-}}{k}$, not at the prescribed *κ*.

### 2.7. Model Parameters, Status, and Assumptions

Table 1 lists every parameter that enters Equations (1)–(9), together with the matrix Poisson ratio used only for the classical Cox comparison of Section 2.3, and gives its numerical value, units, and—most importantly for the interpretation of the results—status for each; all symbols used in this paper are defined in the Nomenclature section at the end of this paper. Three statuses are used. Prescribed parameters are model inputs chosen by the authors, including the checkpoint schedule at which the illustrative summaries are fitted; none of them has been calibrated or fitted to measurement in this work. Geometric parameters are the morphological values that define the nominal operating point at which illustrative evaluations are made. Derived quantities follow from the prescribed and geometric parameters through Equations (1)–(9) and contain no further information. No parameter in this paper has the status of being calibrated, fitted to data, or measured.

The framework rests on the following assumptions, which bound every result reported below. (A1) Inclusions are straight, rigid, monodisperse cylinders; waviness and length polydispersity are neglected [31]. (A2) The matrix is linear elastic, isotropic, and rate-independent; viscoelasticity, hyperelasticity, the Mullins effect, and residual set are not represented. (A3) Deformation is small-strain and uniaxial along the alignment axis. (A4) The orientation distribution is planar Gaussian in the polar angle, with no azimuthal component. (A5) Load transfer follows the classical cylindrical Cox kernel with a single lumped prefactor; the interphase is not resolved. (A6) Interfacial degradation is uniform across the population, scalar, and exponential in cycle number, with no strain-amplitude, frequency, or threshold dependence, and no healing. (A7) The composite is dilute, so inclusion–inclusion interaction and percolation are neglected. (A8) The modulus follows from a closed-form orientation average, not from a solved boundary-value problem; the framework is therefore a closed-form homogenization and not a field-resolved calculation.

### 2.8. Implementation, Internal Verification, and Reproducibility

Every numerical value reported in this paper was obtained by evaluating Equations (1)–(9) in Python 3.11. The complete reference implementation is provided in the Supplementary Materials (Section S2). It uses only the Python standard library: all arithmetic is carried out in decimal floating point at 60 significant digits (results reported to 50), raised to 140 digits for the finite-difference checks of Section 2.4 and Section 3.1, so that the script runs on a bare Python installation and its output does not depend on any third-party numerical library. It implements only the equations printed above, uses no measured data and no previously generated dataset, and prints every entry of Table 2, Table 3 and Table 4, the classical Cox comparison of Section 2.3, and every numerical value quoted in Section 3.1, Section 3.2, Section 3.3, Section 3.4, Section 3.5 and Section 3.6 and the 100-cycle model retention range of Section 4.2 (readings of published curves are tabulated in Table S1 in the Supplementary Materials), including the values marked on Figure 2, Figure 3 and Figure 4, so that each reported number can be recomputed and checked by any reader. It also prints the checks that support Section 2.4, Section 2.5, and Section 3.2: the exact *κ*-independence of the initial local-rate ratio, the *κ*-dependence of the ratio of finite-window apparent rates, the identity *k*_app_ = −ln *Ê*(*N*_max_)/*N*_max_, the window lengths at which $\overset{\text{-}}{k}$ crosses *k*_app_, the strict monotonicity of *k*_app_ in *κ* that makes the calibration inversion of Section 4.4 single-valued, and the dense-grid coefficients of determination quoted alongside the checkpoint values. The figures themselves are generated from the same equations by a second script, provided in the Supplementary Materials (Section S2), which imports the values from the reference implementation and adds NumPy and Matplotlib 3.10.9 only to evaluate the same equations on a plotting grid and to render them.

The reproducibility position of this paper is therefore a single chain, and every link in it is also presented. The model inputs are prescribed and are listed with their values, units, and status in Table 1. Equations (1)–(9) are the complete formulation; no further relation is used anywhere. The reference implementation in the Supplementary Materials (Section S2) implements those equations and nothing else and takes no input file. Every entry of Table 2, Table 3 and Table 4 and every value marked on Figure 2, Figure 3 and Figure 4 is produced by that implementation, and Figure 1, Figure 2, Figure 3 and Figure 4 themselves are drawn from the same equations using the figure script in the Supplementary Materials (Section S2). Previously generated modulus data are not required at any point in this chain, and none is used.

Four internal checks establish that the implementation behaves as the equations require. None of them is a validation; the measurements that would constitute validation are set out in Section 4.4. **Matrix limit.** As *V*_f_ → 0, the reinforcement term of Equation (2) vanishes identically and *E* → *E*_m_ for any orientation state and any cycle count, since *η*_o_ and *η*_l_ are bounded. At the finite volume fractions used for illustration, the same limit appears as *E*_∞_ = (1 − *V*_f_)*E*_m_ = 0.99632 and 0.99005 MPa. **Alignment limit.** As *σ*_θ_ → 0, the director distribution collapses onto the loading axis, *η*_o_ → 1, and Equation (2) returns the maximum reinforcement the closure permits, *η*_l_(*N*)*V*_f_*E*_CNT_. **Decoupling limit.** As *x* → 0, *η*_l_ → *x*^2^/3 → 0 and *E* → *E*_∞_, so the fully cycled state is the constrained matrix. **Sensitivity identity.** Equations (6) and (7) agree with the numerical derivative of ln *η*_l_ to better than 10^−49^, and *S* is confirmed bounded in (0, 1) and strictly decreasing over 10^−4^ ≤ *x* ≤ 10^3^.

**Use of generative artificial intelligence.** Every equation and derivation reported here—including Equations (7) and (8), the compact form of Equation (6), the large-argument asymptote and the monotonicity proof of Section 3.1—is the authors’ own and was not derived with AI assistance, and the authors checked every equation, derivation, and numerical result. The assistance that was used, which covered language editing, independent verification of the relations reported in Section 2.4, Section 2.5, Section 2.6 and Section 3.1, and the writing of the two scripts in the Supplementary Materials (Section S2), is disclosed in full in the acknowledgments statement.

## 3. Analytical Results

All results in this section follow from Equations (1)–(9). Section 3.1 establishes properties of those equations themselves, which hold for any parameter values admitted by the assumptions of Section 2.7. Section 3.2, Section 3.3, Section 3.4, Section 3.5 and Section 3.6 evaluate the same equations at the prescribed and geometric parameters of Table 1: the structural statements they contain are likewise parameter-free, but every numerical value they report is an illustrative calculation in the sense of Section 1 and is identified as such.

### 3.1. The Sensitivity Function and Its Bounds

The sensitivity function of Equation (6) is plotted in Figure 2a. Three exact properties characterize it across its whole range.

**Bounds.** *S*(*x*) ∈ (0, 1) for all *x* > 0. As *x* → 0, the interface is fully decoupled, *η*_l_ ≈ *x*^2^/3, and *S* → 1: the macroscopic rate recovers the full microscopic rate *κ*. As *x* → ∞, the interface is strongly coupled: *η*_l_ sits on the saturated plateau of the tanh and is nearly insensitive to *β*, so *S* → 0.

**Closed-form limits.** For small argument, the expansion of the length efficiency gives 1 − *S*(*x*) = 2*x*^2^/5 + *O*(*x*^4^), so the approach to the fully decoupled limit is quadratic: at *x* = 0.01, the expansion returns 4.000 × 10^−5^ against an exact deficit of 3.99984 × 10^−5^. For large argument, tanh *x* → 1 and sech^2^*x* → 0 exponentially, so Equation (6) reduces to the rational asymptote *S*(*x*) → 1/[2(*x* − 1)]. This form is more accurate than the customary 1/(2*x*): its relative error is 1.0 × 10^−3^ at *x* = 5, 9.1 × 10^−5^ at *x* = 6.31, and below 10^−7^ for *x* ≥ 10. The local rate therefore falls essentially as the inverse of the operating point once the interface is well coupled.

**Monotonicity and uniqueness of the inverse.** *S* is strictly decreasing on (0, ∞), and this follows in closed form. Writing *t* = tanh *x*, Equation (6) is equivalent to the compact form *S* = ½[*xt*^2^/(*x* − *t*) − 1] and differentiating gives d*S*/d*x* = *t*[2*x*^2^ sech^2^*x* − *xt* sech^2^*x* − *t*^2^]/(2(*x* − *t*)^2^). Since *t* > 0 and (*x* − *t*)^2^ > 0 for *x* > 0, the sign of d*S*/d*x* is the sign of the bracket; multiplying the bracket by cosh^2^*x* reduces the claim to the elementary inequality 2*x*^2^ < *x* tanh *x* + sinh^2^*x*. Both halves of that bound are standard. First, tanh *x* > *x* − *x*^3^/3 on (0, ∞) because the difference vanishes at the origin and has derivative *x*^2^ − tanh^2^*x* > 0. Second, sinh^2^*x* > *x*^2^ + *x*^4^/3 on (0, ∞), by the same argument applied twice, using cosh 2*x* > 1 + 2*x*^2^ + 2*x*^4^/3, which is immediate from the Maclaurin series of cosh. Adding the two gives *x* tanh *x* + sinh^2^*x* > 2*x*^2^, so d*S*/d*x* < 0 for every *x* > 0 (Figure 2b). Together with the two limits, this makes *S* a strictly decreasing bijection from (0, ∞) onto (0, 1); a numerical sweep over 10^−4^ ≤ *x* ≤ 10^3^, carried out by the reference implementation in the Supplementary Materials (Section S2), agrees with it. The practical consequence is that the inversion used throughout the rest of this paper is well posed: a single observed value of *k*_loc_/*κ* corresponds to exactly one operating point *x*, so a measured attenuation determines the product *βs* uniquely. Recovering the interfacial rate from that value requires the operating point to be known independently because *x*_0_ = *β*_0_*s* depends on *k*_l_ and *V*_f_ as well as on the aspect ratio; when *k*_l_, *V*_f_, and *s* are specified, as they are in Table 1, the inversion returns the prescribed *κ* exactly. Where *x*_0_ is not known a priori, independent determination of *k*_l_ and *V*_f_ is therefore a precondition, and Section 4.4 indicates how they would be obtained. The uniqueness of the inverse is asserted but not established in earlier shear-lag treatments that invert a fitted decay constant.

### 3.2. Evaluation at the Aligned CNT/PDMS Operating Point

Table 2 evaluates the framework at the nominal operating point of Table 1. At the virgin condition, the shear-lag argument is *x*_0_ = *β*_0_*s* = 6.3116 for the dilute population (*s* = 150.0) and 4.8705 for the concentrated population (*s* = 115.4); *β*_0_ itself differs by only 0.3% between them, so the separation is carried primarily by the aspect ratio. At these points, *S*(*x*_0_) evaluates to only 0.0941 and 0.1290: the initial local decay rate is roughly one tenth of *κ*.

As cycling proceeds, *β*(*N*) decays and *x*(*N*) moves off the saturated branch toward the small-argument regime, so *S*[*x*(*N*)] rises monotonically toward unity (Figure 3a,b). For *x*_0_ = 6.3116, it passes through 0.250 at *N* = 50, 0.584 at *N* = 100, 0.968 at *N* = 200, and 0.9985 at *N* = 300; for *x*_0_ = 4.8705, the corresponding values are 0.346, 0.700, 0.981, and 0.9991. The normalized modulus *Ê*(*N*) is therefore not a pure exponential but a function whose local logarithmic slope increases with cycle number—slow initially, then steepening as the interface approaches full decoupling (Figure 3c).

Fitting a single-parameter exponential to Equation (5) over the four-point schedule returns $\overset{\text{-}}{k}$ = 9.40 × 10^−3^ cycle^−1^ at *x*_0_ = 6.3116 and 11.74 × 10^−3^ cycle^−1^ at *x*_0_ = 4.8705, that is, *κ*/$\overset{\text{-}}{k}$ = 3.28 and 2.63, with half-lives of 73.7 and 59.0 cycles. The single-exponential summary reproduces the exact curve closely at the four checkpoints (*R*^2^ = 0.994 and 0.999 against Equation (5) evaluated there; *R*^2^_adj_ = 0.991 and 0.998). That statistic measures agreement at those four cycle counts and at no others: evaluated instead on a dense grid over the same 0–500 cycle range, the same two summaries return 0.929 and 0.949 because the checkpoints do not sample the steepest part of the trajectory. Every *R*^2^ quoted in this paper is the checkpoint value. An interfacial damage constant read directly off such a fit would therefore be understated by a factor ranging from two and a half to three and a half at aspect ratios typical of aligned CNT bundles.

Two points about these numbers should be kept in view. First, *κ*/$\overset{\text{-}}{k}$ ≈ 3 is not 1/*S*(*x*_0_), which would be 10.6 and 7.8; $\overset{\text{-}}{k}$ is a fit-weighted average over a rising local rate, so it is much larger than the initial local rate. Second, the values in Table 2 are specific to the prescribed *k*_l_ of Table 1 and to the four-point schedule; Section 3.3 and Section 3.4 quantify both dependences rather than leaving them implicit.

**Status of the coefficient of determination.** Every *R*^2^ reported in this paper measures how closely a single exponential summarizes the exact analytical trajectory of Equation (5) at the stated checkpoints. It is a descriptive goodness-of-fit diagnostic for a deterministic curve, not an inferential regression over observations: there is no sampling error, no observational residual variance to estimate, and no competing set of predictors, and the summary has exactly one free parameter, $\overset{\text{-}}{k}$. It is equally a checkpoint statistic rather than a measure of agreement along the continuous trajectory: it is computed from the four values of Equation (5) at *N* = 0, 100, 300, and 500 cycles and carries no information about the cycles between them, where the departure from a single exponential is largest. Quoted instead on a dense grid over the same range, the same summaries return appreciably lower values (Section 3.2), and no *R*^2^ in this paper should be read as continuous-trajectory agreement. The adjusted coefficient of determination is reported beside it throughout—in Table 2 and in Section 3.5—computed in the usual way from the number of checkpoints *n* = 4 and the single fitted parameter *p* = 1, so that *R*^2^_adj_ = 1 − (1 − *R*^2^)(*n* − 1)/(*n* − *p* − 1) = 1 − 3(1 − *R*^2^)/2. Two properties of the adjusted coefficient of determination should be noted. With a fixed schedule and a single free parameter, it is a deterministic affine transformation of *R*^2^, so it orders the summaries identically and adds no independent information; and the penalty it applies is for the number of fitted parameters, not for the sparseness of the schedule, so it does not correct the checkpoint bias described above. It is reported because the penalty makes the descriptive character of both statistics easier to weigh at four points, and for no stronger reason. No *R*^2^ value in this paper is obtained by regression over measured or previously generated data.

### 3.3. Sensitivity to the Prescribed Shear-Lag Prefactor

Because *k*_l_ is prescribed rather than calibrated (Section 2.3), the dependence of the attenuation on it is reported explicitly. *k*_l_ enters the framework at exactly one place, through *x*_0_ = *β*_0_*s*, so the entire sensitivity is a translation along the *x*-axis of Figure 2a. Table 3 evaluates it across that interval and up to the prescribed value.

Three observations follow. First, the direction is unambiguous: a smaller *k*_l_ displaces both populations toward smaller *x*_0_, raises *S*(*x*_0_), and therefore weakens the attenuation. Second, the magnitude is substantial—*κ*/$\overset{\text{-}}{k}$ falls from 3.28 to 1.71 for the dilute population, and from 2.63 to 1.47 for the concentrated one, as *k*_l_ is reduced from 0.042 to 0.019, the lowest value tabulated—so the numerical factor quoted in this paper should not be treated as a material property and is not offered as one. Third, the qualitative conclusion is nevertheless robust across the whole range examined: *κ*/$\overset{\text{-}}{k}$ > 1.4 everywhere, so a fitted macroscopic rate understates the microscopic interfacial rate at every value of *k*_l_ considered, including values inside the classical range. What the choice of *k*_l_ controls is how large the correction is, not whether one is needed.

The form of the map is unaffected by *k*_l_ in a stronger sense as well. Equation (6) contains no reference to *k*_l_, *η*_o_, *V*_f_, or *E*_CNT_; those parameters influence the result only by selecting a point on the fixed curve *S*(*x*). Any statement made about the shape of that curve—its bounds, its limits, its monotonicity, the uniqueness of its inverse—is therefore independent of every prescribed parameter in Table 1.

### 3.4. Dependence of the Apparent Rate on the Measurement Window

Equation (8) makes explicit a dependence that a single fitted constant conceals: the apparent attenuation depends on how long the specimen is cycled. Figure 4b plots *κ*/*k*_app_ against the window length *N*_max_. At *x*_0_ = 6.3116, the exact apparent attenuation is 6.32 over a 50-cycle window, 3.54 over 100 cycles, 1.79 over 200 cycles, and 1.22 over 500 cycles; at *x*_0_ = 4.8705, the corresponding values are 4.54, 2.69, 1.58, and 1.17. The reason is contained in Figure 3b: a short window samples only the slow initial stage, where *S* ≈ 0.1, whereas a long window also averages over the terminal stage, where *S* → 1 and the modulus is already close to its asymptote.

Two practical consequences follow. First, apparent damage rates measured over different cycle windows are not directly comparable, even for nominally identical specimens, and the window must be reported alongside the rate. Second, the attenuation is largest, and the correction therefore most necessary, precisely in the short-window measurements that are most common in practice. The least-squares $\overset{\text{-}}{k}$ of Table 2 falls between the 100- and 200-cycle values of *k*_app_, as Figure 4b shows, because the least-squares fit weights the intermediate checkpoints most heavily; it crosses the exact *k*_app_ at *N*_max_ ≈ 107 cycles for the dilute population and ≈ 103 for the concentrated one. That crossing is numerical and carries no identity: $\overset{\text{-}}{k}$ is not an instance of Equation (8) at any window; the crossing points are fixed by the four-point schedule rather than by a measurement window, and Equation (8) must not be inverted on $\overset{\text{-}}{k}$. The exact *k*_app_ should be used whenever the measurement window is known, which is always, since Equation (8) requires only the normalized modulus at the two ends of that window.

### 3.5. Transfer to Other Filler Geometries: A Design Map

Every geometric input—filler length, effective diameter, and hence the aspect ratio *s* = *L*/*d*_eff_—enters the cyclic-rate mapping only through the dimensionless operating point *x*_0_ = *β*_0_*s*. Re-parameterizing the framework for short carbon fibers, unbundled MWCNTs, or high-aspect-ratio nanowires therefore amounts to relocating *x*_0_ on the fixed curve of Figure 2a and re-evaluating the same sensitivity function. Figure 4a shows this projection for the four-point schedule with *κ* held fixed, together with the initial sensitivity *S*(*x*_0_) for comparison.

The crossover is sharp enough to be read as a design map: $\overset{\text{-}}{k}$/*κ* = 0.90, 0.50, 0.33, and 0.20 at *x*_0_ ≈ 0.98, 3.52, 5.73, and 12.77, corresponding, at the prescribed *k*_l_, to bundle aspect ratios of approximately 23, 84, 136, and 304. At the prescribed *k*_l_, a system with *s* below about 20 may therefore treat a fitted decay constant as the interfacial rate to within 10%, whereas the correction reaches a factor of three at *s* ≈ 136, the third marked point of the map; the corresponding thresholds shift with *k*_l_ as Table 3 shows. Beyond the range marked on the map, a single fitted constant ceases to represent the trajectory, as the next paragraph sets out.

The projection has a definite range of validity, and it is set by the quality of the single-exponential summary rather than by any saturation of the attenuation itself. $\overset{\text{-}}{k}$/*κ* decreases monotonically with *x*_0_ and does not approach a floor: it is 0.223 at *x*_0_ = 10, 0.175 at 20, 0.124 at 100, and 0.059 at 1000. The absence of a floor is a property of the equations rather than of those summaries: *S*(*x*) tends to zero at large argument (Section 3.1), so the exact window-averaged rate of Equation (8), which is *κ* times the cycle-average of *S*, decreases toward zero with it, and does so without appeal to the quality of any fitted constant. What does change is how well a single exponential represents Equation (5). Because the decay does not begin until *x*(*N*) falls to order unity, at *N* ≈ (2/*κ*) ln *x*_0_, a large operating point pushes the onset toward the end of the window: the onset lies at *N* ≈ 149 cycles for *x*_0_ = 10 and *N* ≈ 194 for *x*_0_ = 20. The coefficient of determination of the four-point exponential summary falls correspondingly, from 0.996 at *x*_0_ = 5.7 to 0.963 at 10, 0.937 at 12.8, and 0.892 at 20; the corresponding adjusted values are 0.995, 0.944, 0.905, and 0.838. The design map above is therefore quoted only as far as its last marked point, *x*_0_ = 12.8 (*s* ≈ 304), where *κ*/$\overset{\text{-}}{k}$ = 5.0 and the four-point summary still reproduces the exact curve at its checkpoints with *R*^2^ ≥ 0.93 (*R*^2^_adj_ ≥ 0.90); on a dense grid over the same range the corresponding value is 0.87, and the cutoff is stated in checkpoint terms throughout. That threshold is an author-chosen descriptive cutoff adopted for presentation, and nothing more: it marks where a single exponential ceases to be a useful shorthand for the exact trajectory of Equation (5). It is not a statistical criterion, not a physical threshold, and not a test of the validity of the framework, whose equations hold at every operating point. Beyond that point, a fitted constant loses its meaning: at *x*_0_ = 21.0, the value reached at *s* = 500, the four-point summary returns *R*^2^ = 0.89 (*R*^2^_adj_ = 0.83). The exact window-averaged rate of Equation (8), which requires no fit and remains valid at any operating point, should be used instead. This supersedes any reading of the map as approaching a fixed upper bound on *κ*/$\overset{\text{-}}{k}$: no such bound exists.

Two further caveats bound the projection. *κ* is specific to a given interface chemistry and is held fixed here to isolate the geometric dependence, so transfer to another interface requires the calibration of Section 4.4; and two-dimensional fillers such as graphene nanoplatelets fall outside the cylindrical Cox kernel and the Krenchel average used here and would require a platelet shear-lag formulation.

### 3.6. Illustrative Evaluation of Equation (2)

Table 4 evaluates Equation (2) deterministically at the nominal operating point of Table 1. These values are illustrative calculations, not simulation output or data: they follow from the prescribed parameters by direct substitution and can be reproduced line by line using the reference implementation in the Supplementary Materials (Section S2).

The salient structural property is that Equation (2) is **linear** in the orientation factor. The reinforcement (*E*_0_ − *E*_∞_) = *η*_o_*η*_l_(*x*_0_)*V*_f_*E*_CNT_ scales in exact proportion to *η*_o_, so across the illustrative window *σ*_θ_ = 25–45° the reinforcement can change by at most the ratio of the Krenchel factors, *η*_o_(25°)/*η*_o_(45°) = 0.7439/0.5215 = 1.4265, and Table 4 reproduces that ratio identically for both populations. Any steeper dependence of virgin reinforcement on alignment than this factor of 1.43 cannot be a consequence of Equation (2), whatever the values of the other parameters. This is stated explicitly because it is a hard ceiling that any candidate orientation-scaling law must respect within the present closure; a stronger orientation sensitivity would require additional physics—inclusion interaction, orientation-dependent interfacial area, or a non-affine load path—that this framework does not contain.

The corresponding cyclic behavior is shown in Figure 3c. Because the whole cycle dependence is carried by *η*_l_, the normalized trajectories of the two populations differ only through *x*_0_: within a population, every orientation state follows the same *Ê*(*N*) exactly. A common normalized trajectory across orientation states is therefore a structural property of Equation (2), not an emergent finding, and it is reported here as such.

## 4. Discussion

### 4.1. Physical Interpretation and What the Framework Represents

The framework condenses all interfacial degradation into a single scalar coupling efficiency *η*_int_(*N*) and does not resolve distinct microscopic processes. The mechanisms discussed here are therefore a physical interpretation consistent with that factor, not processes simulated directly: *η*_int_(*N*) is a phenomenological envelope for interfacial debonding, network fragmentation, and the approach to matrix-dominated equilibrium.

In an intact composite, tensile stress applied along the alignment axis is transferred from the compliant matrix into the much stiffer inclusions through a thin interfacial zone of physisorbed and entangled polymer chains, and it is the integrity of this zone—not the bulk properties of either phase—that governs the macroscopic modulus. Cyclic loading is understood to degrade such a zone by irreversible chain slippage at the non-covalent contact, by nanoscale decohesion that isolates inclusion segments from the matrix, and by the consequent attenuation of load transfer [17,18,19,20,25,26,27]. All three act on the Cox length efficiency *η*_l_, and because the macroscopic stiffness scales linearly with *η*_l_, the modulus decays monotonically toward the matrix value. None is individually supported by the present analysis, which contains no mechanism-resolving content.

Within the Cantournet–Diani framework [17,19], each interfacial site dissipates a fixed fraction of its coupling per cycle, so the macroscopic damage inherits the same exponential form with an effective rate equal to the population-averaged site constant—a picture consistent with coarse-grained molecular dynamics on nanoparticle–elastomer systems [21]. The present analysis modifies that correspondence in a way that is not obvious from the site-level argument: the nonlinear shear-lag stage attenuates the observable macroscopic rate well below the site constant, by a factor set by the dimensionless operating point rather than by the site kinetics. A population-averaged site constant inferred by fitting a macroscopic modulus decay is therefore not the site constant unless the attenuation is inverted. That inversion is well posed by the monotonicity result of Section 3.1 and is the practical use of the map.

The fully cycled state follows directly from the form of the prescribed law rather than from any emergent behavior: as *η*_int_ → 0, the reinforcement term of Equation (2) vanishes, and the modulus returns to the constrained matrix value *E*_∞_. Covalent functionalization is known to suppress this softening and to prevent full reversion to the matrix modulus [21,32,33], so the fully cycled state obtained here should be read as the model’s fully decoupled limiting stiffness and not as a lower bound on the cycled-state stiffness of real composites.

### 4.2. Relation to Published Cyclic Measurements

No quantitative comparison between this framework and published cyclic measurements is offered, and the reason is a matter of scope rather than of agreement. The framework is formulated at 1% strain amplitude within a rate-independent, linear-elastic setting (assumptions A2–A3, A6). The available cyclic-tensile data for PDMS/MWCNT micro-composite films, those of Song et al. [22,23], are reported at cyclic amplitudes of 30–70%, a regime in which elastomeric nonlinearity, the Mullins effect, strain-amplitude and strain-rate dependence, viscoelastic relaxation, and accumulated residual set all contribute to the measured change in secant modulus. The present framework represents none of these: *E*(*N*) decays solely through loss of interfacial coupling, and the modeled cyclic response returns to the origin by construction. A retention measured at 30–70% strain and a retention computed at 1% strain are therefore not the same quantity, and treating their numerical proximity as evidence either for or against the model would be a category error. The comparison that follows is therefore one of order-of-magnitude retention only and does not constitute matched-amplitude validation.

The magnitude relation is nonetheless worth stating once because it bears on the prescribed *κ*. Evaluating Equation (2) at the nominal operating point gives a 100-cycle secant-modulus retention *E*(100)/*E*(0) between 0.52 and 0.77 across the illustrative window (reference implementation, Supplementary Materials Section S2), whereas the origin-referred retentions reported after 100 load–unload cycles at 30%, 50%, and 70% amplitude are 0.89, 0.86, and 0.82 [23]. The framework at its prescribed *κ* therefore predicts a greater 100-cycle loss than those measurements show, even though they were made at far larger strain amplitudes. This is a statement about the prescribed value of *κ*, which sets the absolute cyclic timescale and has not been calibrated; it is not a test of the attenuation map, whose sensitivity function *S*(*x*) contains no *κ*.

A second, methodological point limits what any such comparison could establish. The apparent stiffness loss extracted from a published loading curve depends on the reference chosen for the secant modulus. Referred to the origin, the reported hundredth-cycle curves give retentions of 0.89, 0.86, and 0.82; referred instead to the residual-strain offsets of approximately 4.5%, 8.6%, and 13.6% that displace those curves along the strain axis—that is, taken over the active strain range—the retentions become approximately 1.05, 1.04, and 1.01, essentially unchanged after 100 cycles. These are the present authors’ own readings of the published loading curves of [23] and are tabulated in Table S1 in the Supplementary Materials; no published figure is reproduced. In that digitized reading, this re-referencing substantially reduces the apparent change in loading modulus, which suggests that accumulated residual set may account for much of the origin-referred retention change—a mechanism the present framework does not contain. Calibrating *κ* against origin-referred retentions would therefore risk confounding interfacial modulus loss with permanent deformation and would return a constant that does not represent interfacial damage alone. That is a limitation of what a single loading curve can resolve, not a shortcoming of those careful experiments.

**Synthesis.** The comparison with the cyclic measurements of Song et al. [22,23] is therefore contextual, and three statements summarize what it does and does not establish. First, the framework and those measurements occupy different strain regimes and different constitutive settings, so no validation of this framework follows from the published data as reported, in either direction. Second, the attenuation map is unaffected by that mismatch because Equation (6) contains neither *κ* nor the strain amplitude and depends only on the geometric operating point. Third, the discriminating test proposed here is motivated by the structure of Equation (8) and not by any numerical correspondence: for two specimen populations of differing bundle aspect ratio and the same interface chemistry, Equation (8) constrains their apparent rates jointly through a single shared factor exp(−*κN*_max_/2), so eliminating that factor leaves a relation between the two measured rates that contains neither *κ* nor the strain amplitude. It is that relation and not the ratio of the two rates at a stated window that is free of the prescribed damage constant (Section 2.4), and it can be evaluated without resolving the large-strain effects that bound the present formulation. The protocol is set out in Section 4.4.

### 4.3. Engineering Relevance

Two design levers follow from the structure of the framework rather than from the value of *κ*. Where persistent stiffness is required over a service life, acting on the interface—by covalent functionalization of the nanotubes [21,32,33] or by hybrid nanotube/nanoparticle architectures [34]—addresses the mechanism that drives the decay, entering the framework as a reduced *κ* or a nonzero *η*_int_(∞). Where the cycled state, rather than the virgin state, is the figure of merit, the processing cost of alignment returns less than the virgin modulus suggests because the orientation premium enters Equation (2) multiplicatively with *η*_l_ and is therefore consumed as the interface decouples. That second statement applies within the aligned window used for illustration (*σ*_θ_ = 25–45°, *η*_o_ = 0.522–0.744) and is not a recommendation to substitute dispersion-cast material, whose orientation state (*η*_o_ ≈ 0.2–0.375) lies outside it.

The most transferable result is the correction itself. Interfacial damage constants inferred from macroscopic stiffness decay are not comparable across filler geometries or across measurement windows unless the attenuation of Equations (6) and (8) is applied; the design map of Section 3.5 indicates when the correction may safely be neglected and when it dominates the reported number (at the prescribed *k*_l_, *s* ≲ 20 and *s* ≳ 136, respectively). Because Equation (6) depends on the operating point alone and on no material-specific quantity, this applies to any one-dimensional filler/elastomer system that the cylindrical Cox kernel describes, and not only to CNT/PDMS. Cyclically loaded carbon-nanotube and carbon-polymer composites appear in a range of engineering settings in which retention of performance over repeated cycles is the governing design constraint, from compliant structures and flexible electronics [7,8,9,10] to electrochemical energy storage. In the latter, carbon nanotubes are used extensively as conductive additives and as free-standing electrode scaffolds [35], and cycling stability over repeated charge–discharge cycles is a governing figure of merit in the comparative assessment of binary metal-oxide supercapacitor electrodes [36]. Those are settings in which the correction derived here may become relevant; no device-level performance is modeled or claimed in this paper, whose scope is the elastic stiffness of the composite alone.

Two scope exclusions are worth stating explicitly because both are properties of real CNT networks that this mechanical framework deliberately omits. First, the electrical response of a CNT network is sensitive to ambient conditions: the room-temperature gas-sensing response of CNT devices varies measurably with ambient humidity [12], and percolation and tunneling govern conductivity in nanotube composites [29]. Second, electromechanical coupling—the change in resistance that accompanies deformation and interfacial damage—is the basis of the piezoresistive use of these materials [8,9]. Neither effect enters Equations (1)–(9), which describe elastic stiffness only; a coupled treatment would require a conductivity model alongside the mechanical one and is identified as future work rather than approximated here.

### 4.4. Testable Predictions and Calibration Protocol

The framework yields one prediction that constitutes a genuine test of its structure, and several statements that do not. Because the exponential softening form and the value of *κ* are prescribed, neither the exponential trajectory nor the convergence to the matrix asymptote can test the model: both are built in. The attenuation of Equations (6) and (8) is different in kind, since it follows from the shear-lag structure: the function *S* itself is independent of *κ*, of *η*_o_, of *E*_CNT_, and of *V*_f_, which enter at most through the operating point at which it is evaluated. Equation (8) inherits that structure but is not itself free of *κ*: its value depends on *κ* through the window endpoint as well as through the explicit factor, so the test below is framed as a joint constraint rather than as a *κ*-free ratio (Section 2.4).

**The discriminating experiment.** Take two specimen populations of the same interface chemistry but different bundle aspect ratios, cycled over a common window *N*_max_. Equation (8) gives *N*_max_*k*_app,1_ = ln *η*_l_(*x*_0,1_) − ln *η*_l_(*x*_0,1_*u*) and *N*_max_*k*_app,2_ = ln *η*_l_(*x*_0,2_) − ln *η*_l_(*x*_0,2_*u*), with the same *u* = exp(−*κN*_max_/2) in both. The two relations therefore contain a single unknown, and eliminating it leaves one constraint linking *k*_app,1_ and *k*_app,2_ at the known operating points *x*_0,1_ and *x*_0,2_; *κ* no longer appears in that constraint. That constraint is the falsifiable content: the measured pair must lie on the one-parameter locus Equation (8) traces as *κ* varies; equivalently, the value of *κ* recovered from the first population must equal the value recovered from the second. Each inversion is single-valued because *k*_app_ is strictly increasing in *κ* at fixed *x*_0_ and *N*_max_. A departure from that locus, beyond measurement scatter and beyond the tolerance set by the uncertainty in *x*_0,1_ and *x*_0,2_, would be inconsistent with the shear-lag attenuation as formulated here and, within assumptions A1–A8, would count directly against it. What the framework does not provide is a *κ*-free prediction of the ratio *k*_app,1_/*k*_app,2_ at a stated window: that ratio moves with *κ* (Section 2.4) and reduces to the *κ*-free *S*(*x*_0,1_)/*S*(*x*_0,2_) only in the limit of a vanishing window. The test must therefore be stated as the joint constraint or as the agreement of the two recovered values of *κ*, and not as a predicted ratio.

**Calibration protocol.** Quasi-static, fixed-amplitude cyclic tensile tests at the intended operating strain should be run to at least *N* = 500 cycles, with the secant modulus recorded densely over the first ≈150 cycles—where the framework places the steepest change in local rate—and at bounded checkpoints thereafter. At each checkpoint, the modulus should be extracted in both senses distinguished in Section 4.2, referred to the origin and referred to the residual-strain offset, since the difference between them is precisely the residual-set contribution the framework does not represent. Interfacial parameters then follow from nonlinear least-squares fitting of *E*(*N*) = *E*_∞_ + (*E*_0_ − *E*_∞_) exp(−*kN*) to the offset-referred series, first with *E*_∞_ free and then constrained to (1 − *V*_f_)*E*_m_. Agreement between the two fits tests the constrained-asymptote assumption, and the fitted *E*_∞_ supplies the normalization the next step requires. The constant returned by that fit is a least-squares summary of the same kind as $\overset{\text{-}}{k}$, and it is not the quantity to invert. Instead, the normalized modulus *Ê*(*N*_max_) = [*E*(*N*_max_) − *E*_∞_]/[*E*_0_ − *E*_∞_] should be formed at the two ends of the window and the exact apparent rate taken as *k*_app_ = −ln *Ê*(*N*_max_)/*N*_max_, which is Equation (8) evaluated using measurement and requires no fit. Solving Equation (8) for *κ* at the specimen’s operating point *x*_0_ = *β*_0_*s* then converts that rate into a calibrated microscopic constant, replacing the prescribed value. That inversion is single-valued because *k*_app_ increases strictly and without bound with *κ* at fixed *x*_0_ and *N*_max_; the monotonicity result of Section 3.1 establishes the companion uniqueness in *x*, so that a measured attenuation fixes the operating point uniquely once *κ* is known. Repeating the inversion for two or more windows of the same test provides an internal consistency check, since one *κ* must reproduce every window. This is the practical deliverable of this paper. Fitting a single exponential to the whole series and inverting Equation (8) on the fitted constant would conflate the two summaries distinguished in Section 2.5 and is not a valid route to *κ*.

Independent determination of *k*_l_ would remove the largest prescribed uncertainty in the numerical values reported here. Micromechanical measurement of interfacial shear strength on matched bundles, or a field-resolved finite-element calculation of load transfer across the interphase, would each supply it; until then, Table 3 rather than a single value provides the honest representation of what the framework predicts.

## 5. Scope, Reproducibility, and Limitations

### 5.1. What Is Derived, What Is Prescribed, and What Is Illustrative

This paper reports analytical results. Equations (5)–(9) and every property established for them in Section 3.1—bounds, closed-form limits, monotonicity, uniqueness of the inverse, and the exact window-averaged rate—are consequences of Equations (1)–(4) and hold for any parameter values within assumptions A1–A8. They can be checked using a pencil and paper or the reference implementation in the Supplementary Materials (Section S2), which implements only the printed equations.

The numerical values in Table 2, Table 3 and Table 4 and Figure 2, Figure 3 and Figure 4 are illustrative evaluations of those equations using the prescribed parameters of Table 1. They are reproducible in the strict sense that the same inputs return the same outputs, but they are not measurements and are not predictions of the behavior of any particular specimen: two of the inputs that set them—*κ* and *k*_l_—are prescribed rather than calibrated. Statements of the form “the attenuation is a factor of about three” should be read as “Equations (1)–(8) give a factor of about three at the parameters of Table 1”, with the sensitivity of that figure to *k*_l_ given in Table 3 and its sensitivity to the measurement window given in Figure 4b.

Nothing in this paper is validated experimentally, and no claim of validation is made. The internal checks of Section 2.8 establish correct limiting behavior and correct implementation, which is a different and weaker thing. Section 4.4 states the measurement that would constitute a test.

### 5.2. Relation to the Earlier Model-Generated Dataset

An earlier, unpublished numerical study by the present authors generated a set of modulus evaluations over stochastic microstructures at the same nominal operating point, and an earlier version of this manuscript reported statistical properties of that dataset alongside the analytical results. For the present paper, that dataset is historical supporting material and nothing more. The parameters in Table 1 are the nominal inputs of that earlier study, restated here in full and independently of its results (Section 2.1), and no calculation reported here reads it. Its statistical results are not carried forward, and the reason is structural rather than incidental. Equation (2) is exactly linear in the orientation factor, so within the present closure the virgin reinforcement (*E*_0_ − *E*_∞_) can differ across the illustrative window *σ*_θ_ = 25–45° by at most *η*_o_(25°)/*η*_o_(45°) = 1.4265 (Section 3.6); the earlier evaluations differ by substantially more than that. They also carry realization-to-realization scatter that the recorded volume variation does not account for; Equations (1)–(9) cannot produce such scatter, being deterministic in volume fraction and orientation. The earlier evaluations settle above the constrained matrix value (1 − *V*_f_)*E*_m_, which Equation (2) treats as a floor. Those values are therefore not a realization of the formulation documented above: the evaluation that produced them cannot be reconstructed from these equations, and the original implementation is not available. That is a statement about the earlier dataset, not about the present results, none of which use it.

Accordingly, no result, figure, table, or conclusion in this paper depends on that dataset. The variance decomposition, the fitted orientation-scaling exponent, the per-condition fitted decay constants, the master-curve collapse across conditions, and the asymptotic residual reported previously have all been removed, together with the statistical apparatus that supported them, rather than restated in weakened form. What survives is the analytical framework, which never depended on those values. Regenerating a microstructural ensemble from the documented formulation would restore an ensemble-statistical layer on a reproducible footing; it is a well-defined next step and is not required for any claim made here.

### 5.3. Limitations of the Framework

Beyond assumptions A1–A8, four limitations bound the scope of the results.

**Strain regime.** The framework is small-strain and linear-elastic by construction. It does not represent hyperelasticity or any other large-strain nonlinear constitutive behavior, the Mullins effect, strain-amplitude or strain-rate dependence, viscoelastic relaxation, hysteresis, or residual set, all of which are present in real PDMS/MWCNT composites at the 30–70% amplitudes at which they are usually characterized [22,23,37]. It therefore cannot be applied to large-strain cyclic behavior, and the comparison in Section 4.2 is contextual rather than quantitative.

**Prescribed parameters.** *κ* and *k*_l_ are prescribed. The absolute cyclic timescale, including every cycle count quoted in this paper, inherits *κ* and is illustrative until *κ* is calibrated; the magnitude of the attenuation inherits *k*_l_ to the extent quantified in Table 3. Neither prescription affects the form of Equation (6) or any property derived from it in Section 3.1. Equation (8) is a separate case: it is exact, but its value depends on *κ* through the window endpoint as well as explicitly, so an apparent rate cannot be quoted independently of the damage constant that generated it, and ratios of apparent rates are not *κ*-free (Section 2.4).

**Fitted-constant summary.** Equation (6) is exact and Equation (8) is exact, but no general closed-form expression for a least-squares $\overset{\text{-}}{k}$ as a function of *κ*, *x*_0_, and an arbitrary checkpoint schedule is derived. The four-point values of Table 2 are specific to that schedule, and the quality of a single-exponential summary degrades at large *x*_0_ (Section 3.5). This remains to be derived. The limitation is confined to the summary: nothing in the calibration route of Section 4.4 uses $\overset{\text{-}}{k}$, which is reported descriptively and is never inverted.

**Constitutive and morphological simplifications.** Waviness and length polydispersity [31], inclusion interaction beyond the dilute limit, localized microfabrication defects, anomalous stiffening at high cycle counts [24], and electromechanical coupling [8,9,12,29] are all outside the present formulation. Replacing the closed-form orientation average with a field-resolved finite-element treatment of the interphase and admitting a viscoelastic finite-strain matrix are the principal extensions.

## 6. Conclusions

**Contribution.** This paper derives in closed form the relation between the rate at which the interface of a Cox–Krenchel shear-lag composite degrades under cycling and the rate at which its macroscopic modulus falls.

**Main analytical result.** The instantaneous macroscopic decay rate is *k*_loc_ = *κ*·*S*(*x*), where *S* is an exact dimensionless sensitivity function of the single operating point *x* = *βs*. *S* is bounded in (0, 1) and is proved here to be strictly decreasing on (0, ∞), hence a bijection onto (0, 1) with both of its limiting forms available in closed form; the rate apparent over a finite measurement window is likewise exact, equal to *κ* times the cycle-average of *S* over that window. A macroscopic stiffness decay is therefore an attenuated image of the interfacial process, and the attenuation inverts uniquely once the operating point is known.

**Parameter dependence.** Within assumptions A1–A8, the form of the attenuation depends on *x* = *βs* alone: the orientation state, the constituent moduli, and the damage constant do not enter it, while the inclusion geometry, the shear-lag prefactor, and the volume fraction enter only through the value of *x*. At the illustrative operating points, where *S*(*x*_0_) = 0.09–0.13, a constant fitted over a sparse four-point schedule comes out at roughly *κ*/3, and over a 50-cycle window the exact apparent rate understates the prescribed interfacial rate by a factor of 6.32 for the dilute population and 4.54 for the concentrated one. At the prescribed *k*_l_, the design map shows where the correction may be neglected (*s* ≲ 20, within 10%) and where it reaches a factor of three (*s* ≈ 136). The two rates are distinct quantities and are not interchangeable: the window-averaged rate is exact and follows from the normalized modulus at the two ends of the window, whereas the least-squares constant depends on the checkpoint schedule chosen and must not be inverted through Equation (8).

**Limitations.** *κ* and the shear-lag prefactor *k*_l_ are nominal, uncalibrated model inputs rather than calibrated quantities, and neither is a material constant of the CNT/PDMS system, so absolute cycle counts are illustrative and the size of the attenuation is uncertain to the extent set out in Table 3, although it exceeds 1.4 throughout the range tabulated there. The formulation is small-strain and linear-elastic; no experimental validation is claimed, and none should be inferred.

**Experimental calibration.** Calibrating *κ* and *k*_l_ using matched A-CNT/PDMS specimens spanning two bundle aspect ratios, following the protocol of Section 4.4, would convert the map from a correction of principle into a quantitative tool. Those same two populations also carry the falsifiable test of Section 4.4: their exact apparent rates, measured over a common window, must jointly satisfy the two instances of Equation (8) at their respective operating points, both of which contain the same factor exp(−*κN*_max_/2); eliminating it leaves one relation between the two measured rates in which the prescribed damage constant no longer appears and, equivalently, requires both populations to return the same *κ*. A departure beyond measurement scatter would be inconsistent with the shear-lag attenuation as formulated here. The ratio of the two apparent rates at a stated window is not itself free of *κ*; only the initial local rates stand in a *κ*-free ratio, *S*(*x*_0,1_)/*S*(*x*_0,2_) (Section 2.4).

## Figures and Tables

**Figure 1:**
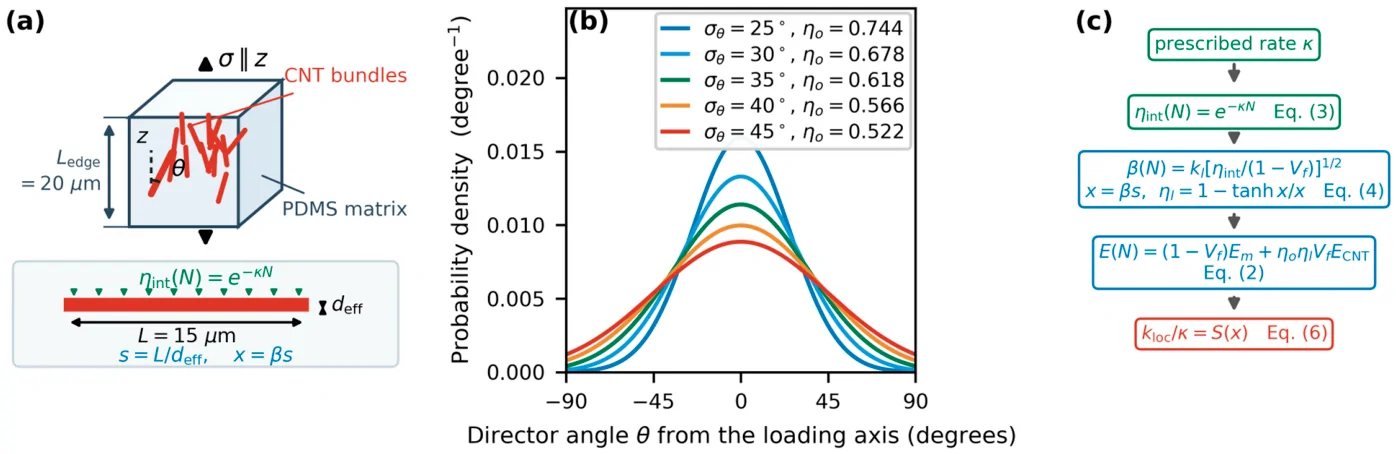
Composite representation and analytical chain. (**a**) Cubic statistical sampling cell of edge *L*_edge_ = 20 µm, drawn as a true cube, containing aligned CNT bundles in a PDMS matrix; bundle lengths, diameters, and positions are schematic and not to scale; both phases are labeled, the loading direction *σ* ∥ *z* is marked at both ends of the cell, and *θ* is the angle of one bundle director from the loading axis. The inset defines the bundle length *L*, the effective bundle diameter *d*_eff_, the aspect ratio *s* = *L*/*d*_eff_, the interfacial coupling efficiency *η*_int_(*N*), and the shear-lag argument *x* = *βs*. No boundary-value problem is solved on the cell. (**b**) Gaussian probability density of the director angle *θ* at the five misalignment levels used for illustration, with the Krenchel factor *η*_o_ from Equation (1) given for each. (**c**) Chain of Equations (2)–(4) linking the prescribed microscopic rate *κ* to the macroscopic modulus *E*(*N*); the sensitivity function *S*(*x*) of Equation (6) is the exact ratio *k*_loc_/*κ* that this chain produces.

**Figure 2:**
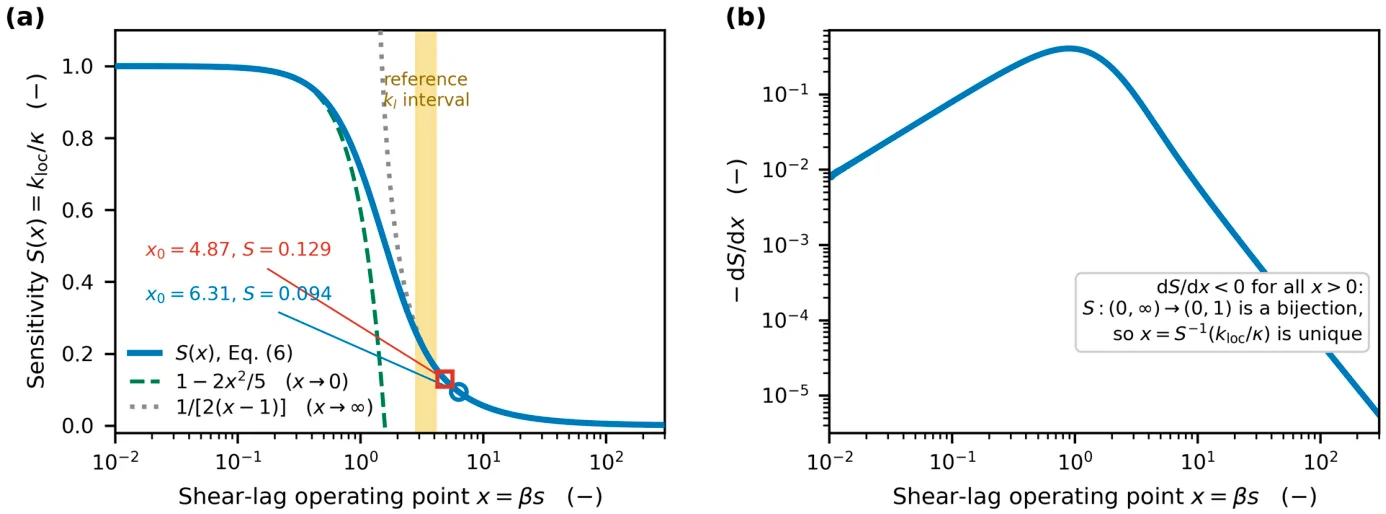
The sensitivity function of Equation (6). (**a**) *S*(*x*) against the dimensionless operating point *x* = *βs* (both dimensionless), with the small-argument form 1 − 2*x*^2^/5 and the large-argument form 1/[2(*x* − 1)]. Open symbols mark the two illustrative virgin operating points *x*_0_ = 6.31 and 4.87; the shaded band spans the operating points that, for the dilute population, the reference interval of Table 3, *k*_l_ = 0.019–0.027, would give. (**b**) Magnitude of the derivative −d*S*/d*x* on logarithmic axes, illustrating the result proved in Section 3.1 that d*S*/d*x* < 0 for all *x* > 0, so that *S* is a strictly decreasing bijection from (0, ∞) onto (0, 1) and its inverse is single-valued.

**Figure 3:**
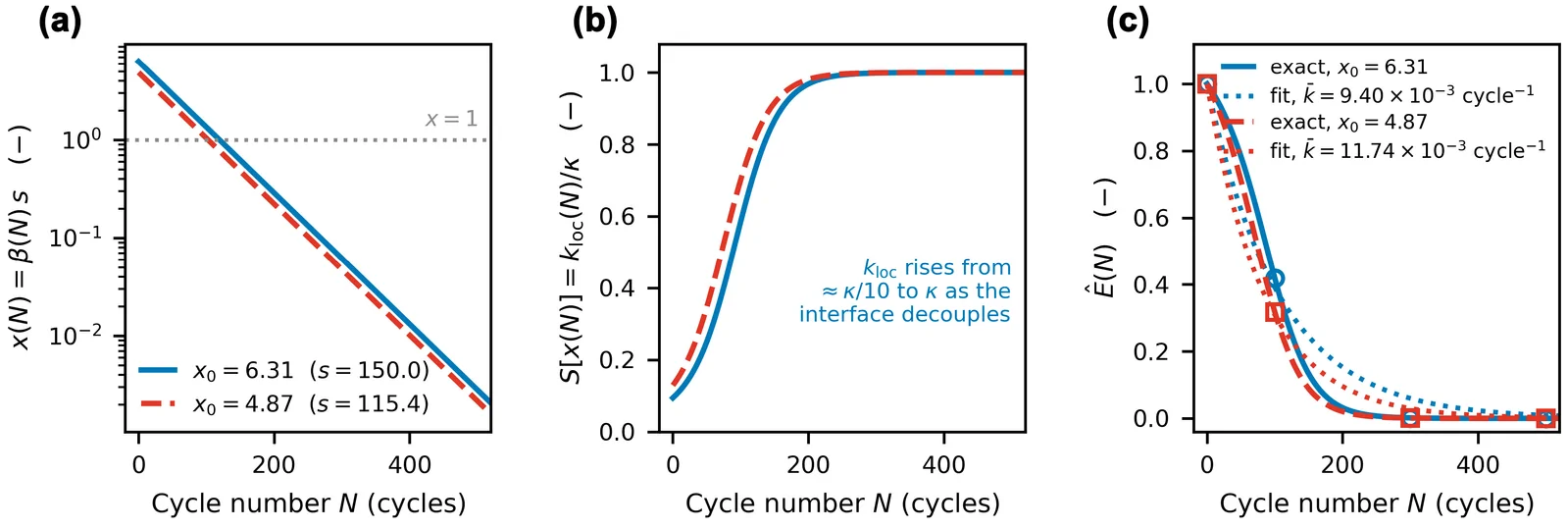
Cyclic evolution predicted by Equations (4)–(6) at the two illustrative operating points. (**a**) Shear-lag argument *x*(*N*) = *β*_0_*s* exp(−*κN*/2) against cycle number, on a logarithmic ordinate. (**b**) Local decay rate normalized by the prescribed microscopic rate, *S*[*x*(*N*)] = *k*_loc_(*N*)/*κ*, rising from about 0.1 at *N* = 0 toward unity as the interface decouples. (**c**) Exact normalized modulus *Ê*(*N*) of Equation (5) (solid and dashed), the four checkpoints of the illustrative schedule (open symbols), and the single-parameter exponential summary fitted to them (dotted). All quantities are dimensionless except the abscissa, in cycles.

**Figure 4:**
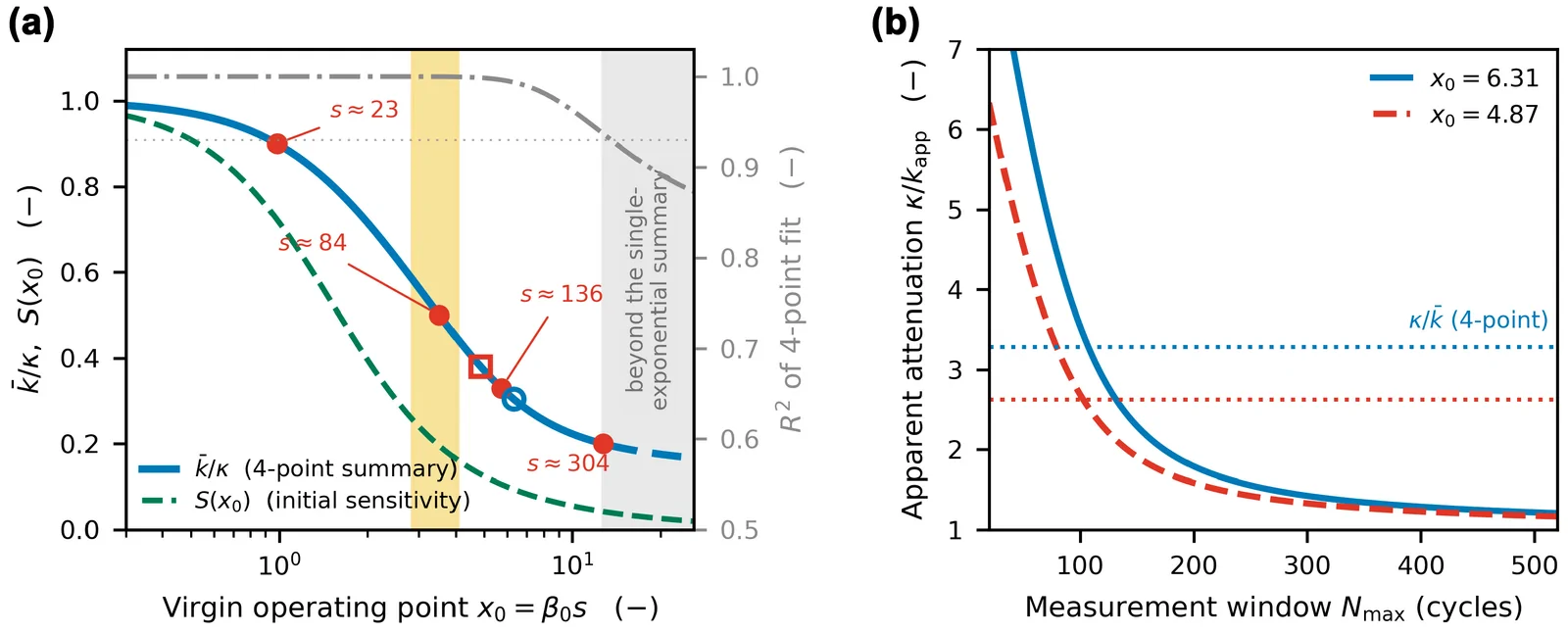
Dependence of the apparent attenuation on geometry and on the measurement window. (**a**) Design map: the four-point least-squares summary $\overset{\text{-}}{k}$/*κ* (solid, left axis) and the initial sensitivity *S*(*x*_0_) (dashed, left axis) against the virgin operating point *x*_0_ = *β*_0_*s*; filled symbols mark $\overset{\text{-}}{k}$/*κ* = 0.90, 0.50, 0.33, and 0.20 with the corresponding aspect ratios at the prescribed *k*_l_; open symbols mark the two illustrative operating points; the shaded band spans the reference interval of Table 3 in *k*_l_. The dash-dotted gray curve (right axis) is the coefficient of determination of the single-exponential summary against Equation (5) at the four checkpoints of the illustrative schedule, which sets the range over which the map may be read. (**b**) Exact apparent attenuation *κ*/*k*_app_ of Equation (8) against the length of the measurement window; dotted horizontal lines mark the four-point least-squares values *κ*/$\overset{\text{-}}{k}$ of Table 2. In (**a**), the map is drawn solid over the range in which the single-exponential summary remains descriptive (four-checkpoint *R*^2^ ≥ 0.93, *x*_0_ ≤ 12.8) and dashed beyond it, where a fitted constant loses its meaning, and the exact Equation (8) should be used instead (Section 3.5). The threshold of 0.93 used for that division is an author-chosen descriptive cutoff adopted for presentation, not a statistical, physical, or model-validity criterion.

**Table 1:** Parameters of the framework, along with units and status. **Prescribed** denotes a model input chosen by the authors and not calibrated, fitted, or measured in this work; **geometric** denotes a morphological value defining the nominal operating point used for illustrative evaluation; **derived** denotes a quantity obtained from the preceding rows through Equations (1)–(9). No parameter in this study has the status *calibrated* or *measured*. Values are shown rounded for display; all evaluations use the unrounded quantities, so that *s* = *L*/*d*_eff_ is 115.3846… for *d*_eff_ = 130 nm.

Symbol	Description	Value	Units	Status
*κ*	Microscopic interfacial-damage rate constant, Equation (3)	3.087 × 10^−2^	cycle^−1^	Prescribed
*k*_l_	Dimensionless shear-lag prefactor, Equation (4)	0.042	–	Prescribed
*E*_m_	Young’s modulus of unfilled cured PDMS	1.00	MPa	Prescribed
*E*_CNT_	Effective bundle modulus entering the shear-lag model	0.4	GPa	Prescribed
*ν*	Matrix Poisson ratio (used only for the classical Cox bound)	0.5 (incompressible limit)	–	Prescribed
*N*	Checkpoint schedule at which the illustrative summaries are fitted	0, 100, 300, 500	cycles	Prescribed
*L*	Bundle length	15	µm	Geometric
*d*_eff_	Effective bundle diameter (dilute/concentrated)	100/130	nm	Geometric
*s*	Bundle aspect ratio, *L*/*d*_eff_	150.0/115.4	–	Geometric
*n*_CNT_	Bundle count in the sampling cell	250/400	–	Geometric
*L*_edge_	Edge length of the cubic sampling cell	20	µm	Geometric
*σ*_θ_	Standard deviation of the Gaussian director distribution	25–45	degrees	Geometric
*V*_bundle_	Bundle volume, π*d*_eff_^2^*L*/4	1.178/1.991 × 10^−19^	m^3^	Derived
*V*_f_	Bundle volume fraction, *n*_CNT_*V*_bundle_/*V*_cell_	0.368/0.995	%	Derived
*η*_o_	Krenchel orientation factor, Equation (1)	0.522–0.744	–	Derived
*β*_0_	Virgin shear-lag parameter, Equation (4)	0.042078/0.042211	–	Derived
*x*_0_	Virgin operating point, *β*_0_*s*	6.3116/4.8705	–	Derived
*E*_∞_	Constrained asymptotic modulus, (1 − *V*_f_)*E*_m_	0.99632/0.99005	MPa	Derived

**Table 2:** Quantities derived from Equations (1)–(8) at the two illustrative operating points of Table 1. *β*_0_ = *k*_l_(1 − *V*_f_)^−1/2^; *x*_0_ = *β*_0_*s*; *S*(*x*_0_) from Equation (6); $\overset{\text{-}}{k}$ is the single-parameter exponential fitted by least squares to Equation (5) over the four-point schedule *N* = 0, 100, 300, and 500 cycles, and *R*^2^ is the coefficient of determination of that summary against Equation (5) at those checkpoints; *R*^2^_adj_ = 1 − (1 − *R*^2^)(*n* − 1)/(*n* − *p* − 1) = 1 − 3(1 − *R*^2^)/2 is its adjusted counterpart at *n* = 4 checkpoints and *p* = 1 fitted parameter. *τ*_½_ = ln 2/$\overset{\text{-}}{k}$. Every entry is reproduced in Section S1 in the Supplementary Materials.

Quantity	Dilute Population (*n*_CNT_ = 250)	Concentrated Population (*n*_CNT_ = 400)	Units
*V*_f_	0.3682	0.9955	%
*s* = *L*/*d*_eff_	150.0	115.4	–
*β*_0_	0.042078	0.042211	–
*x*_0_ = *β*_0_*s*	6.3116	4.8705	–
*η*_l_(*x*_0_)	0.84156	0.79470	–
*S*(*x*_0_) = *k*_loc_(0)/*κ*	0.0941	0.1290	–
*E*_∞_ = (1 − *V*_f_)*E*_m_	0.99632	0.99005	MPa
$\overset{\text{-}}{k}$ (four-point summary)	9.40 × 10^−3^	11.74 × 10^−3^	cycle^−1^
$\overset{\text{-}}{k}$/*κ*	0.3045	0.3804	–
*κ*/$\overset{\text{-}}{k}$	3.28	2.63	–
*τ*_½_ = ln 2/$\overset{\text{-}}{k}$	73.7	59.0	cycles
*R*^2^ of the four-point summary	0.994	0.999	–
*R*^2^_adj_ of the four-point summary	0.991	0.998	–

**Table 3:** Sensitivity of the attenuation to the prescribed shear-lag prefactor *k*_l_, which is not calibrated in this work (Section 2.3). *k*_l_ enters only through *x*_0_ = *k*_l_(1 − *V*_f_)^−1/2^*s*. The reference interval used here, 0.019–0.027, is a rounded interior interval of the classical Cox span computed in Section 2.3; the value used for illustration elsewhere in this paper is 0.042. $\overset{\text{-}}{k}$ is the four-point least-squares summary as in Table 2.

*k*_l_	*x*_0_ (250)	*S*(*x*_0_) (250)	$\overset{\text{-}}{k}$ (250)/10^−3^ Cycle^−1^	*κ*/$\overset{\text{-}}{k}$ (250)	*x*_0_ (400)	*S*(*x*_0_) (400)	$\overset{\text{-}}{k}$ (400)/10^−3^ Cycle^−1^	*κ*/$\overset{\text{-}}{k}$ (400)
0.019	2.855	0.2567	18.00	1.71	2.203	0.3548	21.06	1.47
0.024	3.607	0.1894	15.15	2.04	2.783	0.2653	18.31	1.69
0.027	4.057	0.1626	13.75	2.24	3.131	0.2277	16.88	1.83
0.033	4.959	0.1262	11.56	2.67	3.827	0.1754	14.44	2.14
0.042	6.312	0.0941	9.40	3.28	4.870	0.1290	11.74	2.63

**Table 4:** Illustrative deterministic evaluation of Equation (2) at *N* = 0 using the prescribed parameters of Table 1. These are calculated values, not measurements and not simulation output. The final row records the reinforcement ratio (*E*_0_ − *E*_∞_) between the extreme orientation states, which equals the ratio of Krenchel factors exactly because Equation (2) is linear in *η*_o_.

*σ*_θ_ (degrees)	*η*_o_ (Equation 1)	*E*_0_ (MPa), *n*_CNT_ = 250	*E*_0_ (MPa), *n*_CNT_ = 400
25	0.7439	1.9183	3.3442
30	0.6779	1.8365	3.1353
35	0.6184	1.7627	2.9469
40	0.5662	1.6980	2.7817
45	0.5215	1.6426	2.6403
Reinforcement ratio, 25°/45°	1.4265	1.4265	1.4265

## Data Availability

No new experimental data were created in this study. All results reported here are analytical consequences of Equations (1)–(9) as printed in the manuscript, and every numerical value in Table 2, Table 3 and Table 4 and every value marked on Figure 2, Figure 3 and Figure 4 is reproduced by the reference implementation in the Supplementary Materials (Section S2), which implements only those equations and requires no input data. The values used in the contextual comparison of Section 4.2 are the present authors’ own readings of the published loading curves of reference [23] and are tabulated, with the conditions at which each was read, in Table S1 in the Supplementary Materials. A model-generated dataset produced in earlier, unpublished work by the authors of the present work is described in Section 5.2, together with the respects in which it is not a realization of the formulation presented here. It is historical material only: it is not an input to any calculation reported in this paper, and no result, figure, table, or conclusion presented here depends on it. The analysis code that produced every numerical result and every figure in this paper is provided within this article. The script verify_analytical.py (Section S2 in the Supplementary Materials) implements Equations (1)–(9) and reproduces every entry of Table 2, Table 3 and Table 4 and the classical Cox range in Section 2.3; make_figures.py generates Figure 1, Figure 2, Figure 3 and Figure 4 from the same equations. Neither script reads an input data file. Both were run under Python 3.11; verify_analytical.py requires no third-party package, and make_figures.py additionally requires NumPy and Matplotlib 3.10.9, with the versions used recorded in the accompanying README. The implementation used to generate the earlier dataset described in Section 5.2 is not available, and no part of the present work depends on it; the scripts provided here are an independent implementation of the equations printed in this manuscript and are not a reconstruction of that earlier implementation.

## Supplementary Materials

The following supporting information can be downloaded at https://www.mdpi.com/article/10.3390/ma19194124/s1. Section S1: Closed-form relations used in the supplementary figures; Figure S1: Attenuation of the fitted macroscopic decay rate relative to the prescribed microscopic interfacial damage constant, as a function of the dimensionless shear-lag operating point *x*_0_ = *β*_0_*s*; Figure S2: Local (instantaneous) logarithmic decay rate *k*_loc_(*N*) = *κS*[*x*(*N*)] against cycle number for the two bundle populations, shown with the single fitted global rate $\overset{\text{-}}{k}$ recovered from the same closed form over the four-point schedule (horizontal dotted lines) and the prescribed microscopic constant *κ* toward which the local rate saturates (grey dashed line); Table S1: Digitization audit trail for the experimental points used in the contextual comparison of Section 4.2; Section S2: File manifest.

## Nomenclature

The following symbols and abbreviations are used in this manuscript.

*V*_f_	Effective CNT-bundle volume fraction in the sampling cell (dimensionless; quoted as a percentage)
*n*_CNT_	Number of CNT bundles in the sampling cell (250 or 400)
*L*	CNT-bundle length (15 µm)
*d*_eff_	Effective bundle diameter (100 or 130 nm)
*V*_cell_	Volume of the cubic statistical sampling cell (8 × 10^−15^ m^3^)
*σ*_θ_	Standard deviation of the Gaussian director distribution about the loading axis (degrees)
*θ*	Angle between an individual bundle director and the loading axis (degrees)
*η*_o_	Krenchel orientation factor, ⟨cos^4^*θ*⟩ (dimensionless)
*η*_l_	Cox shear-lag length-efficiency factor (dimensionless)
*η*_int_(*N*)	Interfacial coupling efficiency at cycle *N* (dimensionless)
*k*_l_	Dimensionless shear-lag prefactor (0.042); a nominal, uncalibrated model input, not a measured or transferable material constant
*s*	Bundle aspect ratio, *L*/*d*_eff_ (150.0 or 115.4)
*β*(*N*)	Cycle-dependent shear-lag parameter, *k*_l_[*η*_int_(*N*)/(1 − *V*_f_)]^1/2^ = *β*_0_exp(−*κN*/2) (dimensionless)
*β*_0_	Virgin value of the shear-lag parameter, *β*(0) (dimensionless)
*x*(*N*)	Shear-lag argument, *β*(*N*)*s* (dimensionless)
*x*_0_	Virgin operating point, *x*(0) = *β*_0_*s* (dimensionless)
*k*_loc_(*N*)	Instantaneous local logarithmic decay rate of the modulus, *κ*·*S*[*x*(*N*)] (cycle^−1^)
*k*_app_	Exact apparent decay rate averaged over a measurement window, Equation (8); equals −ln Ê(*N*_max_)/*N*_max_ and requires no fitting (cycle^−1^)
*N*_max_	Length of the measurement window (cycles)
*S*(*x*)	Shear-lag sensitivity function, Equation (6); equals *k*_loc_/*κ*; bounded in (0, 1) (dimensionless)
*E*_m_	Young’s modulus of unfilled cured PDMS matrix (1.00 MPa)
*E*_CNT_	Effective bundle modulus entering the shear-lag model, 0.4 GPa (intrinsic single-tube value ≈1 TPa)
*E*(*N*)	Macroscopic axial Young’s modulus of the composite at cycle *N* (MPa)
*E*_0_	Virgin macroscopic Young’s modulus, *E*(0) (MPa)
*E*_∞_	Constrained asymptotic modulus, (1 − *V*_f_)*E*_m_ (0.99632/0.99005 MPa)
*Ê*(*N*)	Normalized modulus, [*E*(*N*) − *E*_∞_]/[*E*_0_ − *E*_∞_] (dimensionless)
*D*(*N*)	Cyclic damage index, 1 − *Ê*(*N*) (dimensionless)
*N*	Cycle count (cycles)
$\overset{\text{-}}{k}$	Single-parameter exponential summary of the decay over a stated checkpoint schedule (cycle^−1^); descriptive only—not an instance of Equation (8) and not to be inverted through it
*τ*_½_	Half-life of the exponential summary, ln 2/$\overset{\text{-}}{k}$ (cycles)
*κ*	Prescribed microscopic interfacial-damage constant (3.087 × 10^−2^ cycle^−1^)
*G*_m_, *ν*	Matrix shear modulus and Poisson ratio, used only for the classical Cox bound on *k*_l_
*R*^2^	Coefficient of determination of the single-exponential summary at the stated checkpoints; not a measure of continuous-trajectory agreement (dimensionless)
*R*^2^_adj_	Adjusted coefficient of determination of the same summary, 1 − (1 − *R*^2^)(*n* − 1)/(*n* − *p* − 1), with *n* = 4 checkpoints and *p* = 1 fitted parameter (dimensionless)
SVE	Statistical volume element (the cubic sampling cell used here)
CNT/MWCNT	Carbon nanotube/multi-walled carbon nanotube
A-CNT	Aligned CNT architecture
PDMS	Polydimethylsiloxane
CVD	Chemical vapor deposition
