# Supplementary material for: Shear-Lag Attenuation of Interfacial Damage Kinetics in Aligned Carbon Nanotube/Polydimethylsiloxane Composites
Source: Materials (Basel). 2026 Sep 26;19(19):4124. doi: 10.3390/ma19194124 (PMC13665822; doi:10.3390/ma19194124)
Supplement: Supplementary file 1 [file materials-19-04124-s001.zip › materials-4551916-supplementary.pdf]

Supplementary Materials

Shear-Lag Attenuation of Interfacial Damage Kinetics in Aligned Carbon Nanotube/Polydimethylsiloxane Composites

Hassan Syed Tamzid and Jung-Hoon Yun

Contents

- Section S1. Closed-form relations used in the supplementary figures (Equations S1–S4)
- Figure S1. Attenuation ratio $\bar{k}/\kappa$ against the dimensionless operating point x_0
- Figure S2. Local decay rate $k_{loc}(N)$ against cycle number, with the fitted global rate
- Table S1. Digitization audit trail for the experimental points used in the contextual comparison of Section 4.2
- Section S2. File manifest (scripts, figures and dataset)

Section S1. Closed-form relations used in the supplementary figures

Both supplementary figures are evaluated deterministically from the closed-form relations printed in the main text and the prescribed constants of Table 1. Neither figure reads the dataset listed in Section S2, so both are reproducible from the equations alone. The relations are restated here in the notation of the main text; the corresponding main-text equation is given in brackets.

$\eta_l(x) = 1 - \tanh(x)/x$ [Equation (4)]   (S1)

$\eta_{int}(N) = \exp(-\kappa N)$, $\beta(N) = \beta_0[\eta_{int}(N)]^{1/2}$, so $x(N) = x_0 \exp(-\kappa N/2)$ [Equations (3) and (4)]   (S2)

$S(x) = [\tanh(x) - x\,\mathrm{sech}^2(x)]/(2x[1 - \tanh(x)/x])$, $k_{loc}(N) = \kappa S[x(N)]$ [Equation (6)]   (S3)

$\hat{E}(N) = [E(N) - E_\infty]/[E(0) - E_\infty] = \eta_l[x(N)]/\eta_l(x_0)$ [Equation (5)]   (S4)

The fitted global rate is obtained exactly as defined in Section 2.5: a single-parameter least-squares fit of $\hat{E}(N)$ to $\exp(-\bar{k}N)$, performed in linear space over the four sampled cycle counts N = 0, 100, 300 and 500. Because $\bar{k}$ is a fit-weighted average of the monotonically rising local rate over that discrete schedule, it is neither κ nor $\kappa S(x_0)$ alone.

Evaluated at the two bundle populations of this study, the relations above return β_0 = 0.042078 and 0.042211, x_0 = 6.3116 and 4.8705, $S(x_0)$ = 0.0941 and 0.1290, and $\bar{k} = 9.399 \times 10^{-3}$ and 11.743×10^{-3} cycle^{-1}, giving $\bar{k}/\kappa$ = 0.3045 and 0.3804 and $\kappa/\bar{k}$ = 3.284 and 2.629. These agree with the values in Table 2 and Section 3.2 of the main text to the precision at which they are reported.

Figure S1

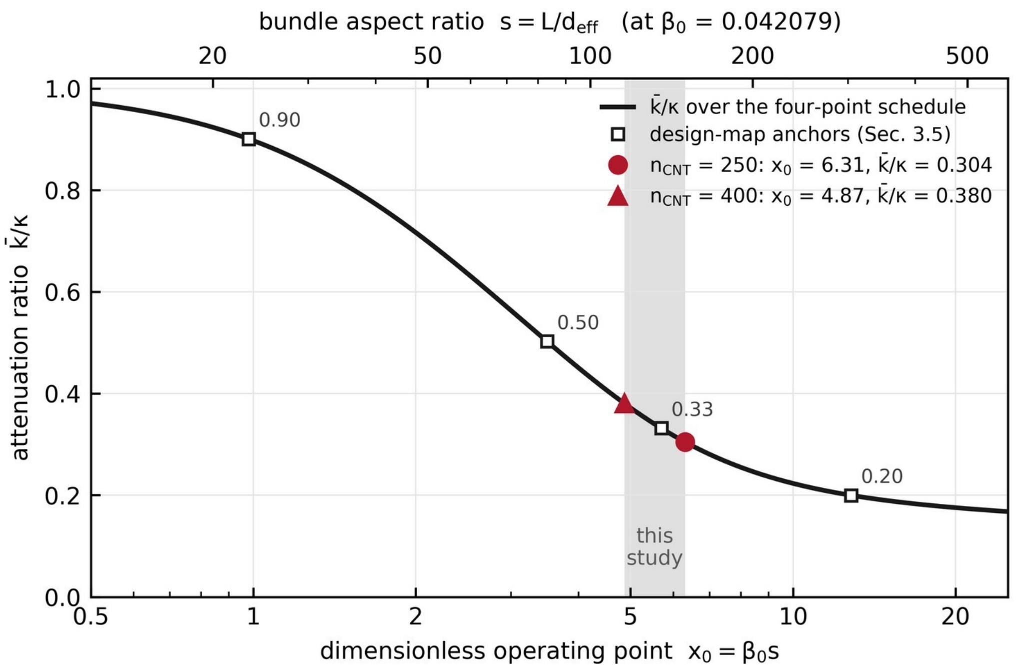

Figure S1. Attenuation of the fitted macroscopic decay rate relative to the prescribed microscopic interfacial damage constant, as a function of the dimensionless shear-lag operating point $x_0 = \beta_0 s$. The curve is Equations (S1)–(S4) evaluated deterministically over the four-point sampling schedule with κ held fixed at 3.087×10^{-2} cycle^{-1}. Open squares mark the design-map anchors quoted in Section 3.5 ($\bar{k}/\kappa$ = 0.90, 0.50, 0.33 and 0.20 at $x_0 \approx$ 0.98, 3.5, 5.7 and 12.8); filled symbols mark the two bundle populations of this study, whose operating points are separated primarily by aspect ratio. The shaded band spans those two operating points. The upper axis converts x_0 to bundle aspect ratio at the dilute-loading value of β_0. A system with s below about 20 may treat a fitted decay constant as the interfacial rate to within 10%, whereas the correction reaches a factor of three at $s \approx 136$. The fitted ratio continues to decrease beyond the plotted range and does not approach a floor (Section 3.5). Generated by figure_S1_attenuation_map.py.

Figure S2

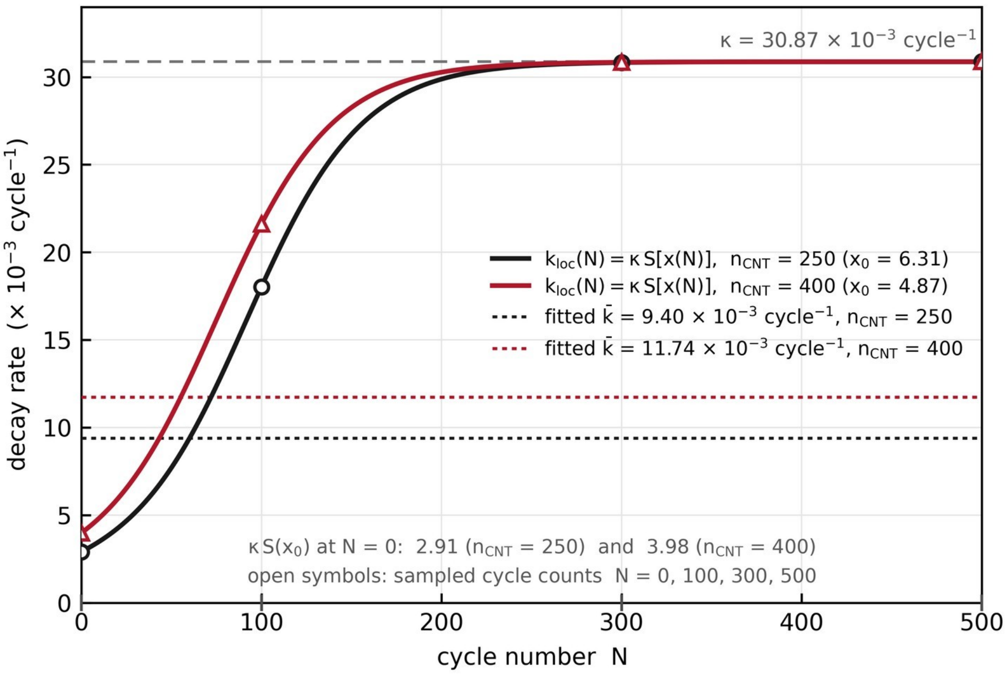

Figure S2. Local (instantaneous) logarithmic decay rate $k_{loc}(N) = \kappa S[x(N)]$ against cycle number for the two bundle populations, shown with the single fitted global rate $\bar{k}$ recovered from the same closed form over the four-point schedule (horizontal dotted lines) and the prescribed microscopic constant κ toward which the local rate saturates (grey dashed line). The local rate begins at roughly one tenth of κ, rises monotonically as $\beta(N)$ decays and S moves off its saturated branch, and exceeds 0.99κ by $N = 300$. The fitted $\bar{k}$ lies well below the curve over most of the window because it is a fit-weighted average dominated by the early, slowly decaying part of the trajectory; this is the origin of the factor-of-three attenuation. Open symbols mark the four sampled cycle counts. The figure is purely analytical. Generated by figure_S2_local_rate.py.

Table S1

Table S1. Digitization audit trail for the experimental points used in the contextual comparison of Section 4.2. Values were digitized from Song et al. [23], Fig. 10(b) (Φ = 5 wt%, $\dot{e}$ = 10 mm/min) under the origin-secant definition of Section 4.2—the stress attained at the cyclic amplitude divided by that amplitude—applied identically to the first and the hundredth load–unload cycle; no published figure or stress–strain curve is reproduced. $R_{orig} = \sigma_{100}/\sigma_1$ is the origin-referred secant-modulus retention; R_{off} is the retention referred to the residual-strain offset, i.e., taken over the active strain range, and follows from the other columns as $R_{off} = R_{orig} \times \varepsilon_a/(\varepsilon_a - \varepsilon_r)$. $\delta\sigma$ is the estimated stress-reading uncertainty (±0.02 MPa) and $\delta\varepsilon_r$ the estimated uncertainty in the residual-strain offset (±0.5%), which dominates the uncertainty of the offset-referred values. The 30% offset-referred value is not treated as evidence, the two metrics being unresolved at that amplitude. Reference numbers follow the reference list of the main text.

Cyclic amplitude ε_a (%)	σ at cycle 1 (MPa)	σ at cycle 100 (MPa)	$\delta\sigma$ (MPa)	Origin-referred retention R_{orig}	Residual strain ε_r (%)	$\delta\varepsilon_r$ (%)	Offset-referred retention R_{off}
30	0.49	0.435	±0.02	0.89	4.5	±0.5	1.05
50	0.79	0.68	±0.02	0.86	8.6	±0.5	1.04
70	1.05	0.86	±0.02	0.82	13.6	±0.5	1.02

Note: Offset-referred retentions are computed from the origin-referred values and residual strains; the 70% entry evaluates to 1.018 and is reported as 1.02.

Section S2. File manifest

The following files accompany this article as supplementary material. Every script implements only the equations printed in the main text, and none reads an input data file.

- **verify_analytical.py**—the reference implementation. It implements Equations (1)–(9) using only the Python standard library (Python 3.11) and prints every entry of Tables 2–4, the classical Cox comparison of Section 2.3 (all eight values), the finite-difference and monotonicity checks of Sections 2.4 and 3.1, every numerical value quoted in Sections 3.1–3.6, and the 100-cycle model retention range of Section 4.2.
- **make_figures.py**—draws Figures 1–4 of the main text from the same equations by importing verify_analytical.py; it additionally requires NumPy and Matplotlib.
- **README**—records the interpreter and library versions used and the commands that regenerate each table and each figure.
- **figure_S1_attenuation_map.py**—generates Figure S1 from Equations (S1)–(S4). Requires numpy, scipy and matplotlib; reads no data file. Writes Figure_S1.png at 600 dpi and Figure_S1.pdf, and prints a verification table of the values quoted in Section 3.5.
- **figure_S2_local_rate.py**—generates Figure S2 from Equations (S1)–(S3). Same dependencies; reads no data file. Writes Figure_S2.png at 600 dpi and Figure_S2.pdf, and prints the tabulated S and k_{loc} values quoted in Section 3.2.
- **Figure_S1.png, Figure_S2.png**—the two supplementary figures as generated, at 600 dpi.
- **CNT_PDMS_DataResult.csv**—the historical dataset from the authors' earlier, unpublished numerical study described in Section 5.2 of the main text: 400 modulus evaluations over 100 microstructural realizations, spanning two filler loadings and five misalignment levels at four cycle counts. It is provided for transparency only; no script reads it, and no result, figure, table or conclusion of the article depends on it. Column meanings are given below.

Column dictionary for CNT_PDMS_DataResult.csv.

Row 1 records the generation date (2026-04-14); row 2 records the prescribed interfacial-damage constant κ = 0.03087 cycle^{-1}; row 3 is the column header; rows 4–403 are the 400 evaluations.

- Cell X / Y / Z [nm]—edge lengths of the cubic sampling cell (20 000 nm each).
- CNT num—bundle count n_{CNT} (250 or 400).
- CNT diameter [nm]—diameter of a single MWCNT (15 nm). This is not the effective bundle diameter (100 nm at n_{CNT} = 250, 130 nm at 400).
- CNT Length [nm]—inclusion length L = 15 000 nm.
- CNT vol [m^3]—mean per-bundle volume for the realization.
- CNT rho / PDMS rho [kg/m^3]—constituent densities (1900 and 970).
- Young's Modulus [MPa]—the evaluated effective axial modulus E.
- Cycle Num—cycle count N (0, 100, 300, 500).
- align set—misalignment level σ_θ in degrees (25, 30, 35, 40, 45).

Use of AI-assisted tools. verify_analytical.py and make_figures.py were written with the assistance of Claude (Anthropic, San Francisco, CA, USA), as disclosed in the Acknowledgments of the main text; the authors checked every equation and numerical result.
